# Conformer Generation Workflows for COSMO-RS Calculations: Are They All the Same?

**DOI:** 10.1021/acs.jcim.6c00606

**Published:** 2026-05-14

**Authors:** André M. M. Gomes, José F. O. Granjo, Paulo J. Costa

**Affiliations:** † BioISI–Instituto de Biossistemas e Ciências Integrativas, Faculdade de Ciências, Universidade de Lisboa, 1749-016 Lisbon, Portugal; ‡ Departamento de Química e Bioquímica, Faculdade de Ciências, Universidade de Lisboa, 1749-016, Lisbon, Portugal; § Hovione FarmaCiência S.A., Estrada do Paço do Lumiar, 86697 Hovione R&D, 1649-038 Lisbon, Portugal

## Abstract

Predicting drug solubility remains a major challenge in the pharmaceutical industry given its central role in formulation development and bioavailability. In this context, the Conductor-like Screening Model for Real Solvents (COSMO-RS) is widely employed to estimate solvation and solubility properties, yet the accuracy of these predictions can strongly depend on the molecular conformations provided as the input. Although this issue has been addressed in previous studies for specific molecules, no systematic evaluation of the influence of conformer-generation methods on the quality of COSMO-RS solubility predictions has been reported using large and chemically diverse data sets. Here, we assess four representative conformer-generation workflows, ranging from simple RDKit-based approaches to more elaborate semiempirical and density functional theory (DFT)-based pipelines, using a curated data set comprising 926 experimental solubility values and 405 free energies of solvation. Despite substantial methodological differences and computational cost, all workflows yield comparable accuracy across the full data set, with mean absolute errors close to one logarithmic unit, and all tend to underperform in the low-solubility regime, which remains a persistent challenge in computational chemistry. We further examined the use of conformer ensembles for representative molecules and compared their performance with solvent-aware single-conformer pipelines. For the cases analyzed, ensemble averaging provides moderate improvements but does not systematically outperform solvent-aware lowest-energy conformer selection. Notably, incorporating solvent information into the lowest-energy conformer identification stage can improve the accuracy for molecules capable of establishing intramolecular interactions, such as hydrogen bonds. Overall, our results provide practical guidance for selecting conformer-generation strategies in high-throughput COSMO-RS applications and highlight solvent-aware workflows as a promising direction to improve the predictive robustness without compromising computational efficiency.

## Graphical abstract

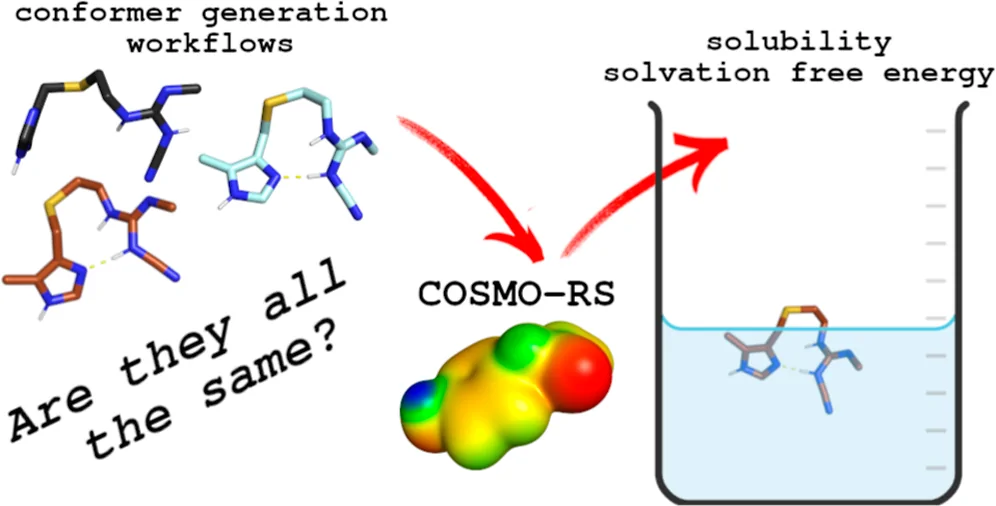

## Introduction

The solubility of active pharmaceutical ingredients (APIs) influences their bioavailability, and understanding this phenomenon is crucial throughout drug and process development, from initial discovery to large-scale manufacturing.^1^ In this context, accurate predictions are essential for optimizing drug formulations tailored to specific delivery systems,^2–4^ even though the prediction of solubility and other thermodynamic properties remains challenging.^5^ Nonetheless, computational tools remain a cost-effective and reliable means for screening drug-like molecules prior to experimental validation. To this end, a wide variety of models are reported in the literature. They span from data-driven approaches, such as the Universal quasi-chemical Functional Group Activity Coefficient (UNIFAC)^6^ or the FASTSOLV models,^7^ to more mechanistic thermodynamic models such as the Statistical Associating Fluid Theory (SAFT) or models based on the Conductor-like Screening Model (COSMO).^8–11^

Among the latter, COSMO-RS (Conductor-like Screening Model for Real Solvents), an extension of COSMO into liquid-phase thermodynamics,^12–14^ stands out for its popularity and ability to integrate quantum mechanical (QM) calculations with statistical thermodynamics, thereby enabling accurate predictions of solubility and solvation properties. Since QM calculations can capture the fundamental electronic and structural properties of molecular systems, being often used to derive descriptors for quantitative structure–activity relationship models,^15^ this synergy allows for a comprehensive understanding of solute–solvent interactions, facilitating the estimation of thermodynamic parameters critical to the design of solvent-based processes and offering valuable insights into the molecular-level mechanisms governing solvation phenomena.^16^ A key aspect of the COSMO-RS approach is the generation of a molecular surface polarization charge density distribution (σ-profile), obtained from a QM calculation in a virtual conductor-like environment. The integration of solute and solvent surfaces enables the calculation of the chemical potential of the solute as a function of the solvent from the local contacts of the surface segments.^17–19^ These results can subsequently be used to estimate interaction energies in solution and activity coefficients, thus enabling the prediction of solubility and partitioning behavior over a range of solvents. Consequently, COSMO-RS is particularly advantageous for solvent screening and design of chemical processes, especially in cases where experimental measurements are challenging, time-consuming, or expensive.^19,20^ However, the accuracy of COSMO-RS predictions can be highly dependent not only on the careful selection of an appropriate QM level of theory, the basis set, and the COSMO parameters but also on the molecular structure (conformation) used for the calculations.

The sensitivity of COSMO-based methods to conformational changes has been addressed over the past few years. This dependence stems from the fact that the conformational diversity of a molecule can lead to significant variations in its electronic structure and surface charge distributions, both of which play a pivotal role in determining predicted thermodynamic properties such as solubility.^21^ Special focus has been placed on the possibility of a molecule forming intramolecular hydrogen bonds, as these interactions strongly affect the σ-profile, with the results critically depending on their correct representation.^22^ Several works have pointed out the effect of including or excluding such conformations in drugs or pesticides,^22^ in atmospheric organic molecules such as citric, tartaric, malic, and maleic acids,^23^ or in mono- and α, ω-dicarboxylic acids.^24^ More recently, for relatively simple molecules such as vanillin and ethyl vanillin, a simple rotation of a hydroxyl group resulted in significantly different values and accuracies of COSMO-RS solubility predictions.^16^ Notice that the presence or absence of such intramolecular bonds depends on the solvent, and thus, accounting for these media differences is also relevant for predictive accuracy.^25^ Accordingly, some authors have pointed out that the use of multiple chemically and mechanistically significant conformations is recommended.^26^ Indeed, results for a given molecular system can be obtained by Boltzmann-weighting the populations of a set of conformers,^9^ with some earlier studies addressing different methods for performing the conformational search, ranging from semiempirical approaches in vacuum to molecular dynamics,^27^ on a small data set of molecules. Nevertheless, for practical purposes such as large-scale database screenings, a conformational ensemble approach is often computationally infeasible, and a single representative conformation is generally employed. These early investigations, despite highlighting that conformational diversity influences the σ-profile, typically addressed only a limited set of case studies or families, rather than performing systematic benchmarking across a diverse chemical space.

Regardless of whether a single conformation or an ensemble of conformers is used, it is clear that the conformer generation step and structure selection are decisive factors that can affect the accuracy of calculated solubilities as well as other chemical properties.^28^ Numerous strategies are described in the literature for generating ensembles of conformers, including exhaustive searches, stochastic sampling, molecular dynamics simulations, and advanced machine learning techniques.^29,30^ These approaches can be further refined by incorporating the distance geometry and knowledge-based structural constraints. Among them, the ETKDG conformer generator,^31^ as implemented in the open-source RDKit Python library,^32^ integrates experimental torsional angle preferences and geometry-based constraints, consistently achieving competitive performance in reproducing crystallographic conformations compared to other state-of-the-art tools^30^ and a favorable balance of accuracy, ensemble size, and runtime on a benchmark for structure-based applications.^33^ Indeed, the cost-effectiveness of ETKDG and similar methods has contributed to its widespread use as a standard approach for conformer generation and a base for further developments, such as machine-learned neural network potential models focusing on reproducing bioactive conformations,^34^ which typically do not surpass traditional algorithms in recovering bioactive conformations relevant to structure-based approaches.^30^ Alternatively, some approaches pursue a more comprehensive exploration of the low-energy chemical space in combination with QM or semiempirical QM-based methods and are thus more computationally demanding. Prominent examples of such approaches are those based on semiempirical tight-binding methods, such as the Conformer-Rotamer Ensemble Sampling Tool (CREST)^35^ or the Global Optimization Algorithm (GOAT)^36^ implemented in ORCA 6.0 software.^37^ Both are tailored to explore the potential energy surface by applying several bias potentials, allowing the system to overcome higher energy barriers and sample diverse conformations. Specifically, GOAT is a refined version inspired by CREST but without requiring metadynamics.^36^

It is clear that the strategies mentioned above differ in their approach: some emphasize a comprehensive exploration at a higher computational cost, while others prioritize computational efficiency and typically rely on data-driven predictions to identify the most relevant conformers.^31,34,35^ However, these methods have been extensively benchmarked in structure-based drug design and bioactive conformation recovery, but this ability is not necessarily transferrable to solubility calculations using COSMO-RS, as structural validation alone may not capture the effects of conformer generation on thermodynamic predictions. Though the impact of the structure in COSMO-RS calculations has been previously addressed,^16,23,24,27^ including workflows explicitly designed to identify relevant solution-phase conformers and their solvent-dependent equilibria,^38^ small data sets of solutes and solvents were generally considered, thus limiting the sampling and significance of the results for large databases of compounds. Most importantly, for the purpose of automating solubility prediction workflows on large data sets, where hand-picking or visually inspecting conformations (e.g., for the presence of intermolecular hydrogen bonds) is not feasible, it is paramount to understand how the choice of a specific conformer-generation method may affect the results and also attempt to provide practical guidance for its selection. Therefore, the scope of our study is to systematically evaluate the impact of various conformer generation methods on the accuracy of solubility values calculated using the openCOSMO-RS^39^ implementation of the COSMO-RS model on a large curated data set comprising 926 data points. We hypothesized that as the conformer-generator methods progressively differ in their a priori accuracy and complexity, they might differently influence the reliability of the calculated properties. The free energies of solvation were also evaluated but for a smaller subset of compounds. Ultimately, this work aims to contribute to the development and optimization of automated workflows that deliver the necessary accuracy and reliability for predicting thermodynamic properties in phase equilibria while also considering computational cost and efficiency, thereby aiding rational drug design and process optimization.

## Methods

### Data Sources

Data were mainly sourced from BigSolDB 2.0,^40^ a public data set of curated solubility values for organic compounds in different solvents, along with values reported in refs 11,41 . From this collection, only those compounds with experimentally reported values for both melting temperature and enthalpy in the NIST Chemistry WebBook^42^ required for solubility calculations (see below) were selected. The overall procedure resulted in 926 data points comprising 185 molecules whose solubilities were experimentally determined across 36 solvents and at different temperatures. The final list of molecules, their ID numbers, and the experimental solubility values, along with the calculated ones, are provided in the Supporting Information (xlsx file). Despite the data set being curated, it should be noted that issues associated with the solid state (e.g., polymorphism, solid–solid transitions, cocrystal formation, solvent inclusion, and variations in experimental protocol) can contribute to uncertainty in the reported experimental solubilities. However, such uncertainties are intrinsic to large data sets and reflect the conditions under which predictive methods such as COSMO-RS are typically applied, particularly in early-stage studies where solid-state information is not yet fully characterized. This reflects typical real-world application scenarios rather than an idealized benchmarking setting. As all workflows are evaluated on the same data set, these effects are not expected to introduce systematic bias in the comparative analysis.

**Figure 1:**
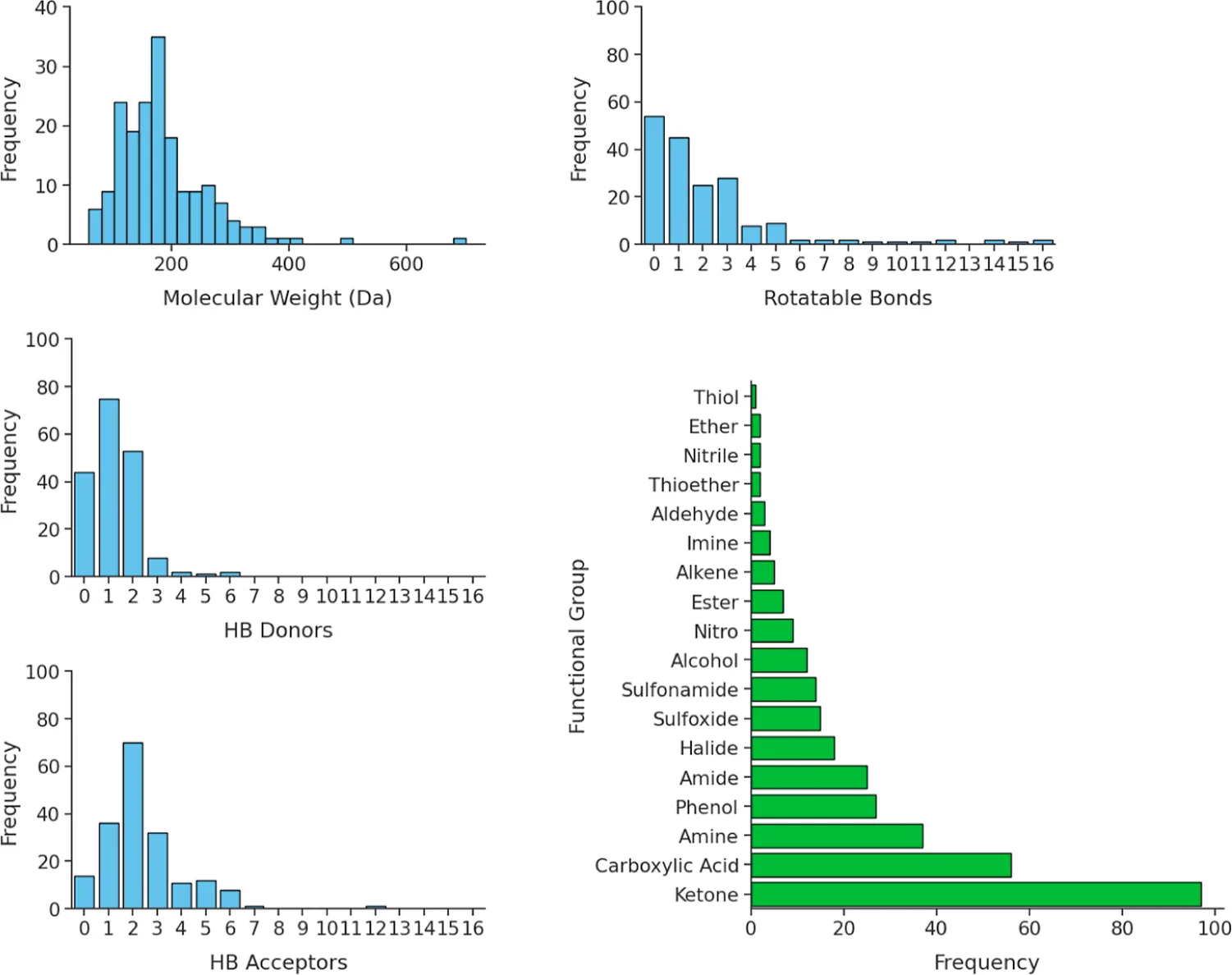
Characterization of the data set comprising 185 molecules, including the distribution of molecular weight, number of rotatable bonds, hydrogen-bond (HB) donors and acceptors, and the occurrence of relevant functional groups.

The final molecular data set encompasses compounds with a diverse yet drug-like chemical profile (Figure 1). The molecular weights are predominantly distributed between 100 and 400 Da, suggesting moderate-sized molecules within the typical drug-like range, although there are a few exceptions with significantly higher molecular weights, exceeding 500 and even 700 Da. Most compounds have fewer than five rotatable bonds (∼87%), with the maximum number of torsions observed for stearic acid and nonadecane (16 rotatable bonds). While a larger population of highly flexible molecules would enable a more systematic exploration of the limits of conformer-generation performance, for practical purposes, our distribution can be considered representative of a subset of relatively rigid drug-like molecules encountered in pharmaceutical pipelines. Our results are therefore not expected to be directly transferable to highly flexible systems such as macrocycles or peptides, for which conformational space exploration is substantially more complex and whose subsequent quantum chemical calculations are computationally demanding, thereby limiting their inclusion in high-throughput benchmarking studies. We also note that the maximum number of rotatable bonds in our data set is consistent with the fact that only a small fraction of approved small-molecule drugs exhibit more than 16 rotatable bonds, as reported in ChEMBL.^43,44^ Although molecules with five or more rotatable bonds represent a minority of the compounds considered, the corresponding number of data points remains significant (on the order of hundreds), enabling a meaningful assessment of conformer-generation performance across different flexibility regimes. In terms of hydrogen-bonding potential, most molecules possess no more than three hydrogen-bond donors and acceptors, well within Lipinski’s rule of five criterion. A functional group analysis reveals a high prevalence of ketones, carboxylic acids, and amines, while groups such as thiols and ethers are relatively rare.

To further evaluate the performance of the methods on related experimental observables, experimental values of the Gibbs free energy of solvation (Δ*G*_solv_) were retrieved from ref 45, which contains a compilation of data from multiple sources. To ensure the overlap between both properties, only solute pairs that are present in our solubility data set were considered, resulting in a total of 405 Δ*G*_solv_ data points. This subset is also provided in the Supporting Information (xlsx file).

### Conformer Generation Methods

The molecules in our data sets, represented using a 2D SMILES notation, were subsequently sanitized and converted into RDKit^32^ molecule objects. The 3D molecular structures were then obtained using automated conformer generation approaches, as explained below, each progressively varying in a priori accuracy and complexity. The first workflow, named ***RDKit***-***Direct***, used the version 3 of the stochastic experimental torsion with knowledge-based distance geometry (ETKDGv3)^31^ method, as implemented in RDKit (version 2024.09.04).^32^ In this procedure, a single conformer was generated and subsequently optimized using the Merck molecular force field (MMFF94).^46^ Notice that this conformation might not necessarily correspond to the lowest-energy structure, and, as such, this pipeline can be considered a baseline null model, due to its simplicity.

The second workflow, referred to as ***RDKit***-***DFT***, also employed the RDKit-ETKDGv3 method, as previously described. However, in this case, conformer ensembles are generated for each molecule. The number of generated conformers was determined based on the number of rotatable bonds, following the heuristic criteria based on ref 47 and used in the openCOSMO-RS conformer pipeline^48^ (see below), whose filtering criteria were also applied in the following steps. Succinctly, after generation, the conformers were filtered to retain only those within an energy window of 6 kcal mol^–1^ relative to the lowest-energy structure. Next, the selected conformers were clustered using a 1.0 Å RMSD cutoff, and the lowest-energy conformer from each cluster was selected. These representatives were then subjected to a geometry optimization step using the MMFF94 force field, and only the lowest-energy conformer from this step was retained. Finally, a DFT geometry optimization was performed at the BP86/def2-TZVP level of theory in an ideal conductor (ϵ = ∞) using the Conductor-like Polarizable Continuum Model (CPCM) in ORCA^37^ (version 6.0).

The third workflow, denoted as **GOAT**, uses the global optimization algorithm (GOAT),^36^ conformer generator, implemented in the ORCA software.^37^ The algorithm, inspired by basin hopping, simulated annealing, and taboo search, without resorting to molecular dynamics, generated a series of conformers that were optimized at the extended tight-binding GFN2-xTB^49^ level. From these, the lowest-energy conformation was selected and subsequently optimized at the DFT/BP86/def2-TZVP level in an ideal conductor, as in the previous workflow.

Finally, the fourth workflow, termed ***openCOSMO***-***RS_c_p***, used the openCOSMO-RS conformer pipeline^48^ implemented in the ConformerGenerator.py script.^50^ Succinctly, the procedure begins with the generation of 3D conformers using the RDKit distance geometry embedding method and proceeds through sequential optimization steps, starting with GFN2-xTB geometry optimizations in water using the analytical linearized Poisson–Boltzmann (ALPB) model, followed by DFT optimizations carried out in both the gas phase and the CPCM model in an ideal conductor, further comprising an additional refinement step at the DFT/BP86/def2-TZVP­(-f) level. This pipeline is recommended by the openCOSMO-RS framework, and further details can be found in ref 48.

Overall, the different workflows, schematically summarized in Figure 2, were selected for comparison either by their use of distinct conformer generation algorithms and/or variations in the number of geometry refinement steps, leading to different computational costs and eventually accuracy in the prediction of solubility. All workflows used in this study involve at least one geometry optimization step, which with a high degree of probability yields a reasonable local minimum on the potential energy surface. Accordingly, and following the reference ***openCOSMO***-***RS_c_p*** pipeline,^48^ full vibrational frequency analysis was not carried out. Therefore, while explicit frequency verification could provide additional formal confirmation of true minima, it is not expected to systematically alter the comparative conclusions of this large-scale benchmarking study.

**Figure 2:**
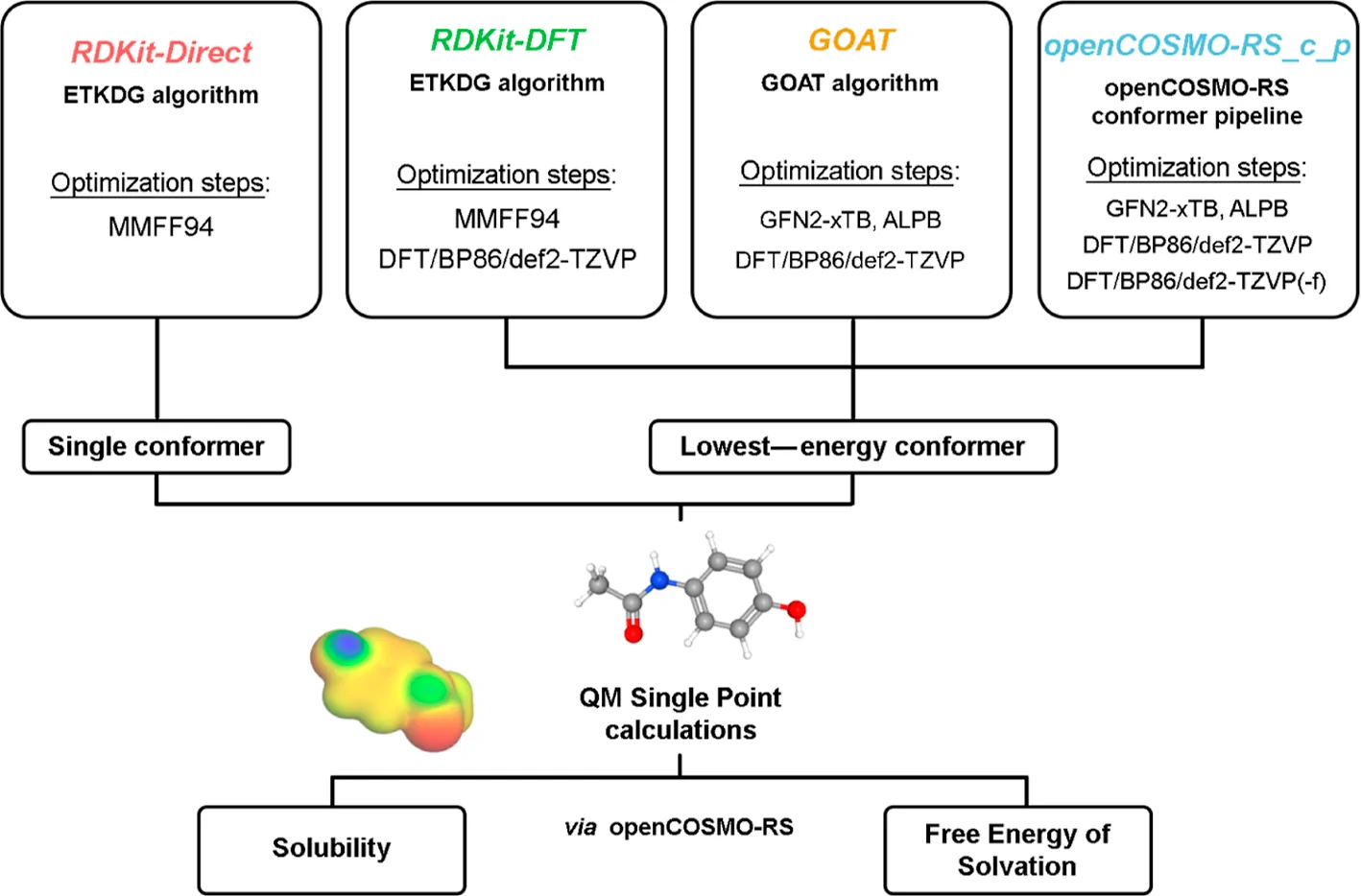
Schematic representation of the four workflows used to generate the lowest-energy 3D molecular structures, highlighting the integration of the respective computational methods.

### Solubility and Free Energy of Solvation Calculations

For each workflow described above, only the single lowest-energy conformer was considered, in line with the reference openCOSMO-RS 24a^48^ implementation, unless explicitly stated otherwise, and used as input for a QM single-point energy calculation. These calculations were run using the Becke exchange and Perdew correlation (BP86) functional^51,52^ and the triple-ζ valence polarized basis set with diffuse functions (def2-TZVPD), in the gas phase and in a perfect conductor, as required in the openCOSMO-RS parametrization.^39^ These single-point DFT calculations and resulting molecular surface charge densities (σ-profiles) were processed with the openCOSMO-RS python package,^39,53^ using parametrization version 24a.^48^ Note that no attempt was made to further refine this parametrization scheme. We observe a reduced overlap between the chemical space of the present data set and that used for parametrization of openCOSMO-RS 24a, as assessed by projection onto the first two principal components of the MACCS or Morgan fingerprints (Figure S1). This observation might be relevant when applying a fixed parameter set across distinct chemical spaces.

The infinite dilution activity coefficients of the solid component in the liquid phase (γ_solid_) were computed iteratively, from which the solubility of the solute (*x*_solid_, mole fraction of the solid in the liquid phase) can be determined using eq 1.^54^

1 $$\ln\left(x_{solid}\gamma_{solid}\right)=\frac{\Delta_{m}H}{R}\left(\frac{1}{T_{m}}-\frac{1}{T}\right)+\frac{\Delta_{m}C_{p}}{R}\left(\frac{T_{m}}{T}-\ln\frac{T_{m}}{T}-1\right)$$

Here, *R* represents the ideal gas constant, while *T* denotes the absolute temperature of the system in *K*. This equation also incorporates experimental parameters of the pure solute, namely, the melting temperature (*T*_*m*_, in K), the melting enthalpy (Δ_*m*_ *H*, in J mol^–1^), and the difference in heat capacity upon melting (Δ_*m*_ *C*_*p*_). Considering the relatively small contribution of the heat capacity change term compared to the melting enthalpy term, and acknowledging that Δ_*m*_ *C*_*p*_ is typically not readily measurable or available in the literature, the Δ_*m*_ *C*_*p*_ term was neglected, which leads to eq 2.^54^

2 $$\ln\left(x_{solid}\gamma_{solid}\right)=\frac{\Delta_{m}H}{R}\left(\frac{1}{T_{m}}-\frac{1}{T}\right)$$

Note that for solubility predictions, the fluid-phase contributions are combined with terms arising from solid-state thermodynamics, which might introduce a source of uncertainty, as already mentioned above. The solubility values were then converted to mg mL^–1^ using the solvent density at 298.15 K (see xlsx in Supporting Information). Notice that experimental solubility data correspond predominantly to temperatures within 298K ± 5 K. For this narrow range, this approximation is not expected to introduce significant deviations in the calculated values.

The Gibbs free energy of solvation (Δ*G*_solv_) was directly obtained from the final BP86/def2-TZVPD single-point calculations using ORCA 6.0 (*COSMO-RS­(solvent)* keyword). In this type of calculation, ORCA calls the openCOSMO-RS executable and computes Δ*G*_solv_ as the difference between the Gibbs free energies in solution and in the gas phase, derived from the same underlying COSMO-RS sigma-profiles. More details on this topic can be found in the ORCA manual.^55^

### Analysis and Confidence Interval Estimates

To assess the accuracy of our models, we primarily report the Mean Absolute Errors (MAEs, eq 3) and the Root Mean Square Errors (RMSEs, eq 4)­

3 $$MAE=\frac{1}{N}\sum_{i=1}^{N}\left|y_{i}^{\exp}-y_{i}^{pred}\right|$$

4 $$RMSE=\sqrt{\frac{1}{N}\sum_{i=1}^{N}{\left(y_{i}^{\exp}-y_{i}^{pred}\right)}^{2}}$$

where *y*_*i*_^*exp*^ and *y*_*i*_^pred^ denote the experimental and predicted values for the sample *i*, respectively, and *N* represents the number of data points (explicitly disclosed on each analysis). The MAE serves as a straightforward measure of average prediction error without considering the direction of the errors, whereas the RMSE provides a more sensitive measure of error magnitude by penalizing larger deviations. Given the wide range of solubility values in mg ml^–1^ units, a logarithmic (base 10) transformation was applied to both experimental and predicted solubility values, while for the Δ*G*_solv_, the analysis was performed on the absolute values (in kcal mol^–1^).

To quantify the uncertainty associated with the MAE and RMSE of a given method *X*, we estimated the lower (*L*_*X*_) and upper (*U*_*X*_) limits of their 95% confidence intervals using eqs 5 and 6.^56–58^

5 $$L_{X}={\left(MAE/RMSE\right)}_{X}\left(1-\sqrt{1-\frac{1.96\sqrt{2}}{\sqrt{N-1}}}\right)$$

6 $$U_{X}={\left(MAE/RMSE\right)}_{X}\left(\sqrt{1+\frac{1.96\sqrt{2}}{\sqrt{N-1}}}-1\right)$$

Note that this estimation is valid only for sufficiently large sample sizes (*N* >8) and that the resulting confidence intervals are asymmetric. Since the prediction errors are strongly correlated due to the use of the same COSMO-RS model and reference data, pairwise statistical significance was assessed using a composite uncertainty that explicitly accounts for correlation. Two methods *A* and *B* were considered statistically different (95% confidence level) when the absolute difference in their MAE (or RMSE) exceeds the correlated composite uncertainty (eq 7),^56–58^

7 $$\left|{MAE}_{A}/{RMSE}_{A}-{MAE}_{B}/{RMSE}_{B}\right|>\sqrt{L_{A}^{2}+U_{B}^{2}-2r_{AB}L_{A}U_{B}}$$

where *r*_AB_ is the Pearson correlation coefficient between the per-molecule prediction errors of the two methods. Notice that owing to this correlation, even small differences in error metrics may be statistically significant.^58^

For the Spearman rank correlation coefficient, approximately 95% confidence intervals (*CI*_ρ_) were computed using a Fisher *z*-transformation (eq 8), where ρ is the observed Spearman correlation coefficient and *N* is the sample size. For sufficiently large sample sizes (e.g., *N* ≥25), this transformation yields an approximately normally distributed population.^59,60^

8 $$CI_{\rho }=\tanh\left[\tanh^{-1}\left(\rho \right)\pm \frac{1.96}{\sqrt{N-3}}\right]$$

Finally, to assess structural similarity between molecular conformations, the root-mean-square deviation (RMSD) between atomic coordinates (excluding hydrogen atoms) was evaluated using a Python program publicly available in ref 61. This tool provides built-in functionality for atom reordering and structural alignment, allowing for accurate RMSD calculations while accounting for differences in atomic ordering between the compared molecules.

## Results and Discussion

### Comparison of Conformer-Generator Workflows

Before addressing the impact of molecular geometry on solubility and solvation free-energy calculations, we first examine how each workflow explores different regions of conformational space (when available). We stress that this analysis is not intended to assess which conformer-generation algorithm provides better agreement with experimental structures, nor which one yields the lowest-energy conformer. Instead, our goal is to compare how different workflows sample conformational space relative to one another. To this end, we calculated the cross root-mean-square deviation (RMSD) of the heavy-atom positions (in Å) between the final conformations produced by the four workflows. The resulting average values are depicted as a matrix in Figure 3 (top), together with the distribution of RMSD values using ***RDKit***-***Direct*** as a reference (Figure 3, bottom). The mean RMSD values between methods are approximately 0.6 Å, which is reasonable for molecules bearing a moderate number of rotatable bonds. It is worth noting, however, that even for very small RMSD values, differences in the calculated solubilities can be substantial, as illustrated by the case of vanillin, which has only two rotatable bonds.^16^ In this example, a simple hydroxyl group rotation corresponds to an RMSD of 0.0 Å (or 0.4 Å if hydrogen atoms are included) between the two rotamers yet leads to very large differences in COSMO-RS predictions.^16^ Overall, taking ***RDKit***-***Direct*** as a reference, it is evident that the different conformer-generation workflows can produce distinct structures. This is reflected by the skewed distribution of larger RMSD values, indicating increasing structural divergence between the different methods and the null model. For instance, ***GOAT*** is able to sample structures with higher RMSD values, whereas the distribution for ***RDKit***-***DFT*** is not so broad, as expected. These differences can be more evident if one examines individual molecules of varying size and flexibility. For a small and relatively rigid molecule such as paracetamol (ID 175 in the xlsx file, see Supporting Information) possessing a single rotatable bond, the conformations are very similar, regardless of the workflow (Figure S2, upper panel), but can still reach an RMSD of ≈0.6 Å (***RDKit***-***Direct*** vs ***GOAT***). On the contrary, for a more complex molecule such as glyburide (ID 174 in the xlsx file, see Supporting Information), bearing eight rotatable bonds, a larger variability on the final generated conformations is observed leading to high RMSD values (Figure S2 lower panel), ranging from 1.6 Å to 2.5 Å. These discrepancies are especially pronounced in complex molecules with multiple rotatable bonds, underscoring the distinct outcomes produced by different conformer-generation methods.

**Figure 3:**
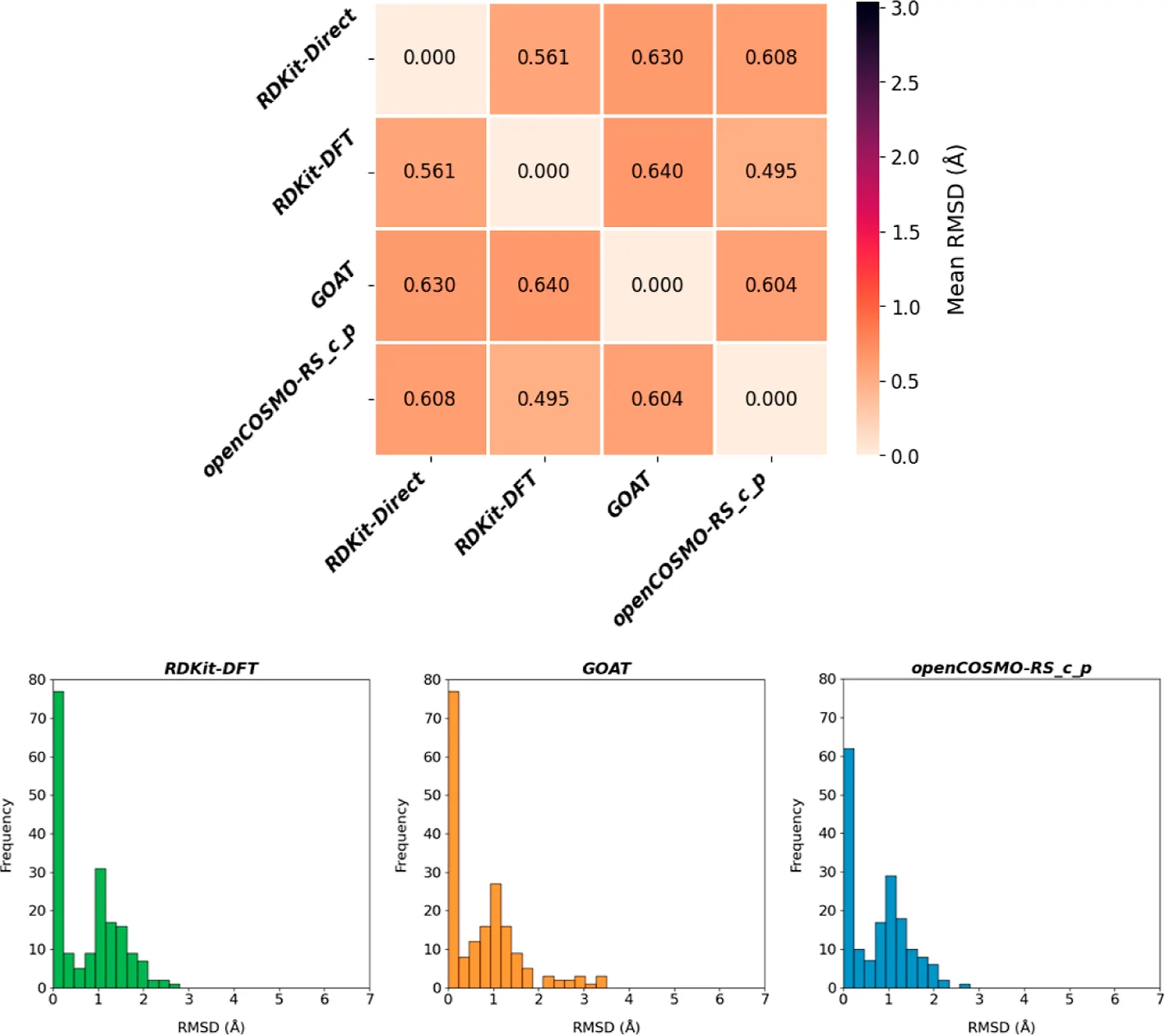
Matrix of the mean cross RMSD values (Å) between the final conformers obtained from the different workflows (top), along with the distribution of the RMSD values for each workflow, using ***RDKit***-***Direct*** as a reference (bottom).

### COSMO-RS Solubility Predictions on the Full Data Set

Having established that the four workflows can lead to different geometries, we then assessed their impact on calculated solubility values on the full data set of molecules (926 data points) by plotting the calculated log_10_ solubility values as a function of the log_10_ experimental values along with the corresponding MAE and RMSE values for each workflow (Figure 4). The points predominantly occupy the upper quadrant of the parity plots, highlighting a uniform positive shift, evidencing a general overestimation that is workflow independent. This is further illustrated by the distribution of the calculated deviations for each conformer generation workflow (Figure S3), which reveals a similar behavior across all pipelines: an almost unimodal distribution around the parity line along with a consistent shift toward positive values. This persistent bias suggests that all workflows tend to yield predictions that overestimate the corresponding experimental values, regardless of the specific conformer generation pipeline employed. Nevertheless, all workflows demonstrated reasonable COSMO-RS predictive capability, with MAE values around 0.90–0.95 log units. Despite its simplicity, ***RDKit***-***Direct*** surprisingly achieves the best performance on the full data set, whereas the other workflows yield comparable results but at a higher computational cost. A pairwise statistical comparison using correlated composite uncertainty (Table S1) further indicates that, although the MAE values are similar, the superior performance of ***RDKit***-***Direct*** may still be statistically significant due to error correlation. However, for practical high-throughput screening purposes, no single workflow clearly emerges as optimal. Another feature of these findings is that all workflows tend to underperform in the low-solubility region, as evidenced by the dispersion of data points in that area. This highlights a current challenge of predictive thermodynamic models, such as COSMO-RS, in accurately predicting the solubilities of poorly soluble solutes. We will further discuss this point in the following Section.

**Figure 4:**
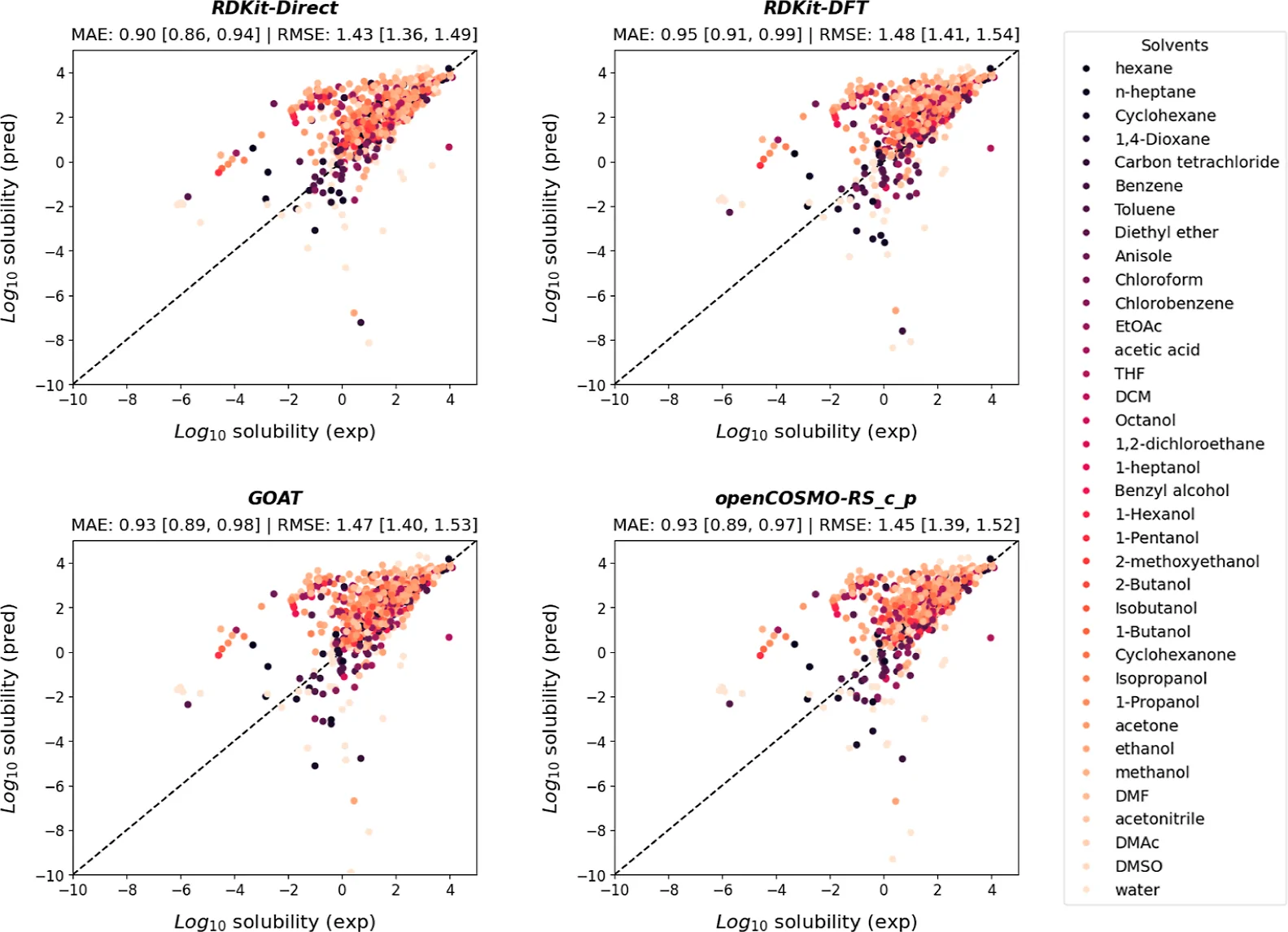
Parity plots comparing the predicted (pred) and experimental (exp) solubility values (log_10_) for the different conformer generation workflows across the total solubility data set (*N* = 926 data points). Dashed black lines represent perfect parity (1:1) and are not fitted to the data. Data points are color-coded by a qualitative solvent polarity gradient, from dark (less polar) to light (more polar). The MAE and RMSE values are reported with the associated 95% confidence intervals. Pairwise statistical comparison of MAEs and RMSEs using correlated composite uncertainty is reported in Tables S1 and S2, respectively.

We also investigated whether the nature of the solvent is related to the prediction quality, but no clear correlation was observed between prediction errors and solvent polarity. This is qualitatively illustrated by the color gradient of the data points in Figure 4. To further explore this aspect, we analyzed the impact of the conformer-generation workflow on the MAE of COSMO-RS-predicted solubilities for each solvent (Figure 5; see Figure S4 for RMSE values). Despite the variation in the number of data points (i.e., solutes) available for each solvent, which can naturally affect the MAE values, only 1-pentanol, 1-propanol, and water exhibit MAEs well above 1.0 log units. Other protic solvents, such as methanol, ethanol, or 1-butanol, show MAE values below this threshold; therefore, no general trend can be established. Overall, since no clear relationship is observed between the error magnitude and solvent polarity, factors beyond polarity alone, such as specific solute–solvent interactions, are likely contributing to the observed variations. The consistent overall trends and solvent-wise distributions suggest that, while methodological differences impact the accuracy to some extent, further refinement of the openCOSMO-RS 24a parameters may be necessary, particularly in the low-solubility range and regardless of the conformer generation workflow used. However, such optimization was not explored in this study. The reduced overlap between our data set and the one used to parametrize openCOSMO-RS 24a might also contribute to this observation (S1).

**Figure 5:**
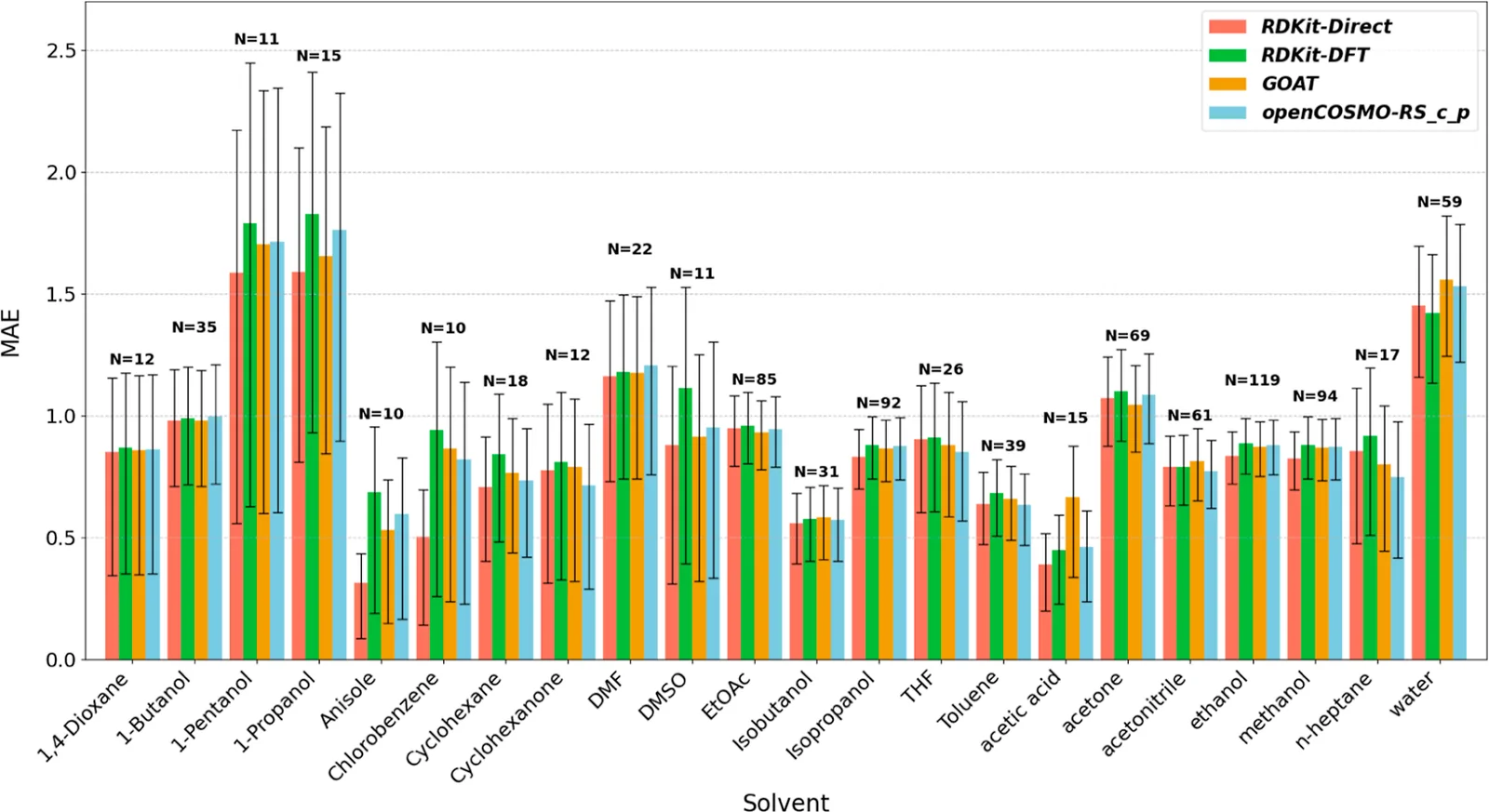
Mean Absolute Error (MAE) for solubility (in log_10_ mg mL^–1^) between calculated and experimental values for each solvent and each conformer generation workflow. The number of data points (*N*) used for the calculation of the MAE for each solvent is also shown. Only solvents with *N* >8 were included in the plot, thus allowing us to show the associated 95% confidence intervals.

With respect to the comparison between conformer generation workflows, the MAE values for a given solvent typically fall within a similar range across all methods. Accordingly, no workflow consistently outperforms the others for specific solvents, although method-dependent differences can be observed in some cases. For instance, for anisole and DMSO, ***RDKit***-***DFT*** stands out as the poorest-performing approach, despite its similarities with ***RDKit***-***Direct***, highlighting that even subtle differences in molecular geometry can lead to noticeable changes in the calculated solubility values. In contrast, for acetic acid, ***GOAT*** yields the largest MAE values.

### Comparative Performance across Solubility and Flexibility Regimes

The previous results on the full data set indicate that, for high-throughput screening purposes, none of the tested workflows clearly emerge as optimal, although ***RDKit***-***Direct*** exhibits a slightly better overall performance. In addition, all workflows struggle to accurately predict poorly soluble compounds. Therefore, a more thorough analysis is needed to address this limitation and to potentially highlight subtle advantages of specific conformer generation pipelines across distinct solubility ranges or numbers of available torsions. For this purpose, a useful strategy is to calculate the MAE, RMSE, or other performance metrics over progressively larger subsets of the data, ordered according to the variable of interest (e.g., experimental solubility or number of rotatable bonds). In practice, the performance metric is computed by sequentially including data points starting either from the lowest values (e.g., lowest solubility or flexibility) or, conversely, from the highest values, until the full data set is considered. Accordingly, the values at the far right of the plots correspond to the overall metrics across the complete data set, while the left-hand side reflects the error associated with the most challenging regimes. This cumulative representation allows us to assess how the prediction performance evolves across different regions of chemical space and whether differences between workflows become more pronounced in specific regimes.

We started this analysis by looking at the behavior of the different conformer generation methods on different solubility regimes by plotting the cumulative MAE as a function of decreasing or increasing experimental solubility (Figure 6). See Figure S5 for a similar plot using the RMSE. On the decreasing solubility plot (Figure 6, left), the high-solubility region exhibits peaks in MAE, though these lack statistical significance owing to limited sampling (i.e., small number of data points and no confidence intervals). The best performance, corresponding to a minimum MAE of about 0.3 (log_10_ mg mL^–1^), is achieved when the analysis is restricted to compounds whose experimental solubility is above 10^3^ mg mL^–1^ (*N* = 252 data points). Curiously, at this region, ***RDKit***-***Direct*** performs worse than the remaining conformer-generation pipelines. As less-soluble compounds are incorporated into the analysis, the MAE values steadily increase, eventually reaching a plateau corresponding to the final values reported in Figure 4. If we restrict the analysis to compounds with solubilities above 1 mg mL^–1^ (*N* = 807), a threshold represented as a dashed line in the plots, the MAE remains below 0.8 (log_10_ mg mL^–1^). Notice that despite the close proximity of the MAE values, their differences can still be statistically significant at the 95% confidence level (Table S3). If we now perform the inverse analysis, i.e., consider only compounds whose solubility is below 1 mg mL^–1^, represented on the left of the dashed line in Figure 6, right (*N* = 120 data points), the error is markedly higher (MAE >2) indicating a significant degradation in predictive accuracy regardless of the conformer generation workflow employed. In this situation, as the more soluble compounds are added to the analysis, the MAE drops, eventually converging to the final values of ≈0.9 reported in Figure 4. Still, even in the low-solubility regime, the fast and simple ***RDKit***-***Direct*** method (in red) slightly stands out by consistently yielding lower MAE values, which, despite not being large, could still be significant (Table S4).

**Figure 6:**
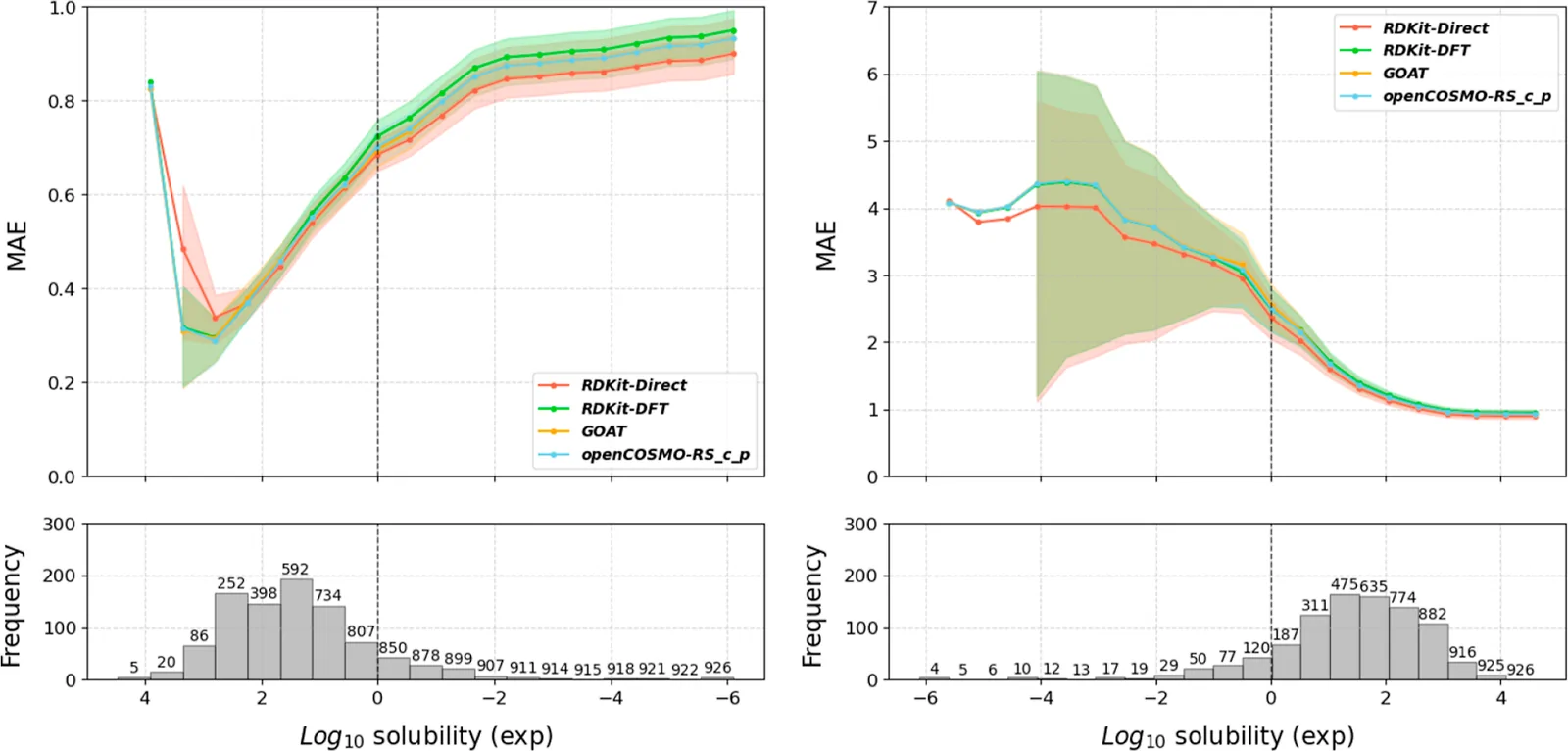
Cumulative MAE (in log_10_ mg mL^–1^) for each conformer-generation pipeline, calculated over progressively larger subsets of experimental solubility data. Subsets are ordered from high to low (left panels) or from low to high (right panels) solubility. Shaded areas represent the associated 95% confidence intervals (for cases when the number of data points *N* >8). The bottom panels display the bin populations together with the cumulative *N* used in each successive MAE calculation. A uniform bin width of 0.5 log units was employed. The dashed vertical lines indicate the 1 mg mL^–1^ solubility threshold.

To investigate the effect of molecular flexibility on the performance of the workflows, we also calculated the cumulative MAE over progressively larger subsets of molecules ordered by the number of rotatable bonds (Figure 7). Starting with the increasing number of rotatable bonds (Figure 7, left), the MAE suddenly increases when molecules with more than 2 rotatable bonds are added to the data set. Still, by sequentially including more data points with increasing flexibility, the curve corresponding to ***RDKit***-***Direct*** stays consistently below the other methods. Indeed, considering compounds with fewer than 5 rotatable bonds (highlighted by the dashed line, 800 data points), the difference between ***RDKit***-***Direct*** and the other methods can be significant (Table S5). While it is true that our data set comprises a large fraction of molecules with fewer than five rotatable bonds, and it is not surprising that a simple approach such as ***RDKit***-***Direct*** performs reasonably well, the limited conformational flexibility of these molecules would be expected to lead to similar performance across all workflows, rather than systematically favoring the simplest one.

**Figure 7:**
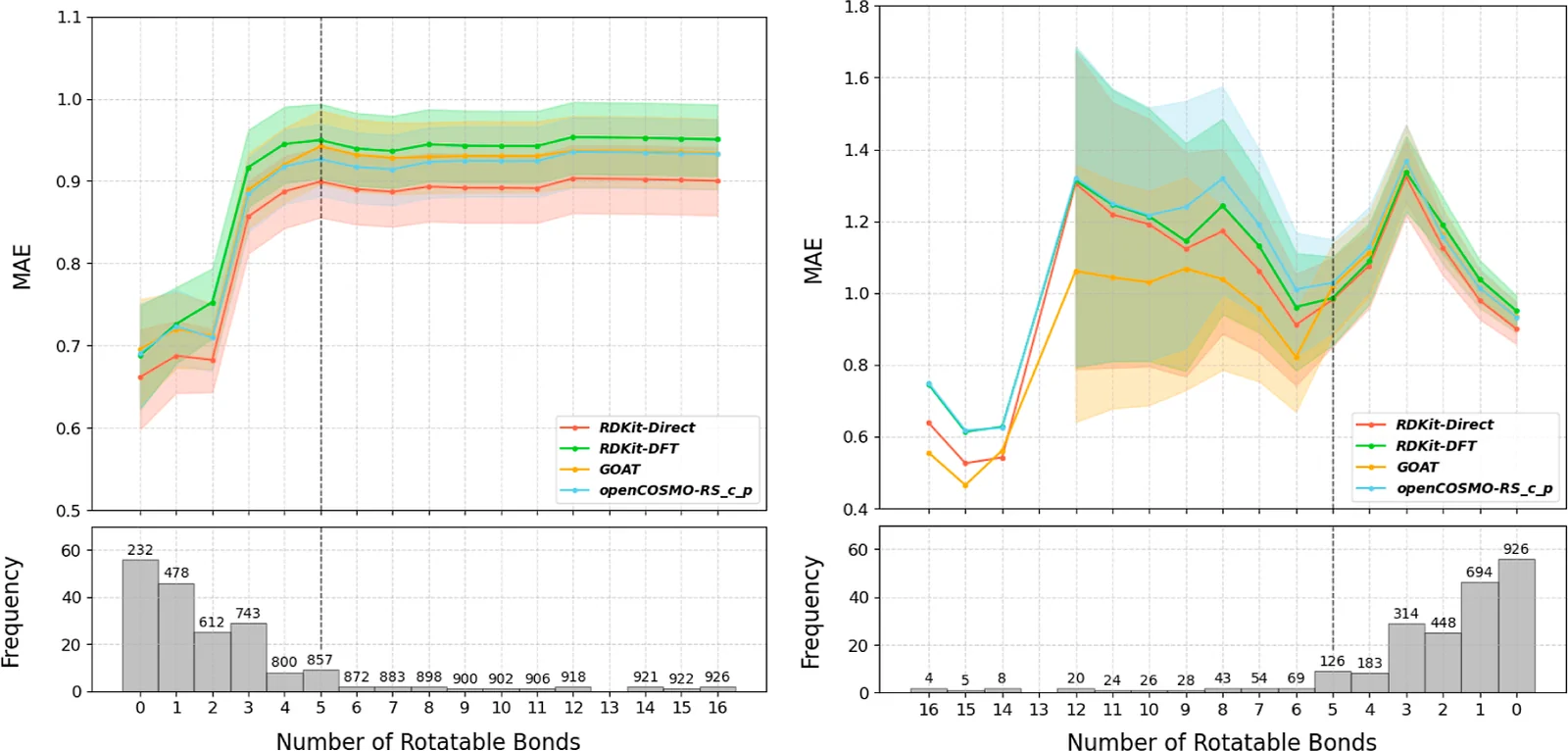
Cumulative MAE (in log_10_ mg mL^–1^) for each conformer-generation pipeline, computed over progressively larger subsets of molecules ordered by the number of rotatable bonds, from low to high (left) and from high to low (right). Shaded areas represent the associated 95% confidence intervals (for cases when the number of data points *N* >8). The bottom panels display the bin populations together with the cumulative *N* used in each successive MAE calculation. A uniform bin width of 0.5 log units was employed. The dashed vertical lines indicate a 5 rotatable bond threshold.

Our data can also be used to construct a specific subset comprising more flexible molecules bearing ≥5 rotatable bonds. Such a subset remains sufficiently populated (*N* = 126 data points, Figure 7, right). Notice also that this population, despite being a fraction of the full data set, still allows for meaningful statistics and is larger than typical sets used to address conformation flexibility in the context of COSMO-RS calculations. Indeed, on the decreasing flexibility plot (Figure 7, right), we observe a crossing point at 5 rotatable bonds (see Table S6 for pairwise comparisons). In the high-flexibility regime (left of the dashed line of Figure 7, right), the ***GOAT*** workflow outperforms the remaining methods, with the largest difference observed for molecules with 12 rotatable bonds (*N* = 20 data points). Notice, however, that while the number of rotatable bonds seems an important factor for choosing more complex methods such as ***GOAT***, it should not be regarded as the sole surrogate for sensitivity to conformational effects in solubility predictions. Indeed, as mentioned earlier, the COSMO-RS calculations on vanillin (two rotatable bonds) are highly sensitive to a single bond rotation.^16^

Despite the observed moderate quantitative agreement described above, for practical purposes of high-throughput screening, it would also be important to assess how these workflows behave in ordering compounds by solubility. Indeed, such rank-based predictions can still provide valuable guidance in solvent screening, particularly in the early stage or when experimental data are scarce. Figure S7 depicts the same concept of using progressively larger subsets of the data, but this time, Spearman’s correlation coefficient is employed in place of MAE. This nonparametric measure of rank correlation is particularly useful to evaluate how well the relationship between two variables can be described using a monotonic function based on their ranks rather than their raw values. Since no linearity is assumed, this coefficient allows us to assess whether, as one variable increases, the other also increases (or decreases), though not necessarily at a constant rate (i.e., whether the correct order or ranking can be predicted). The coefficient ranges from −1 (perfect inverse ranking) to 1 (perfect agreement), with 0 indicating no correlation. In our specific case, it provides a robust assessment of each workflow’s ability to reproduce the correct ordering of solubility values relative to the experimental data, regardless of the magnitude of individual prediction errors. When analyzing the plots in the direction of decreasing solubility (Figure S7, left), a plateau situated slightly above 0.6 is reached, indicating a moderate ability to reproduce the experimental rank order with no striking differences observed between the chosen workflows. However, for the low-solubility regime, and considering the increasing solubility plot instead (Figure S7, right), the ability to predict the order as measured by Spearman’s rank correlation coefficient does not reach 0.5 for the less soluble compounds (<1 mg mL^–1^, *N* = 120 data points), thus indicating that regardless of the workflow used, the correlations are not strong enough for reliable rank prediction.

### Solvation Free Energies

Since the Gibbs free energy of solvation (Δ*G*_solv_) can also be obtained from COSMO-RS calculations, a parallel analysis of this thermodynamic property was conducted using the subset of molecules described in the Methods section. Given that Δ*G*_solv_ is also dependent on the input conformation, this approach additionally enables assessment of the impact of different conformer generation methods. Figure 8 depicts the parity plots between the predicted and experimental values for all workflows. The results indicate that slight differences are found between the pipelines, as highlighted by the errors (reported in kcal mol^–1^), with the more complex ***GOAT*** and ***openCOSMO***-***RS_c_p*** workflows showing a slightly better overall accuracy (MAE ≈1.2 kcal mol^–1^; see Table S7 for pairwise statistical comparison). Here, and contrary to what was observed in solubility prediction, the ***RDKit***-***Direct*** exhibits slightly higher errors, particularly pronounced for specific solvents such as cyclohexanone (Figures S8 and S9). This trend is consistent with improved estimation of conformer energies obtained via DFT-based geometry optimization (i.e., relaxation), thereby enhancing the accuracy of solvation free-energy predictions.

**Figure 8:**
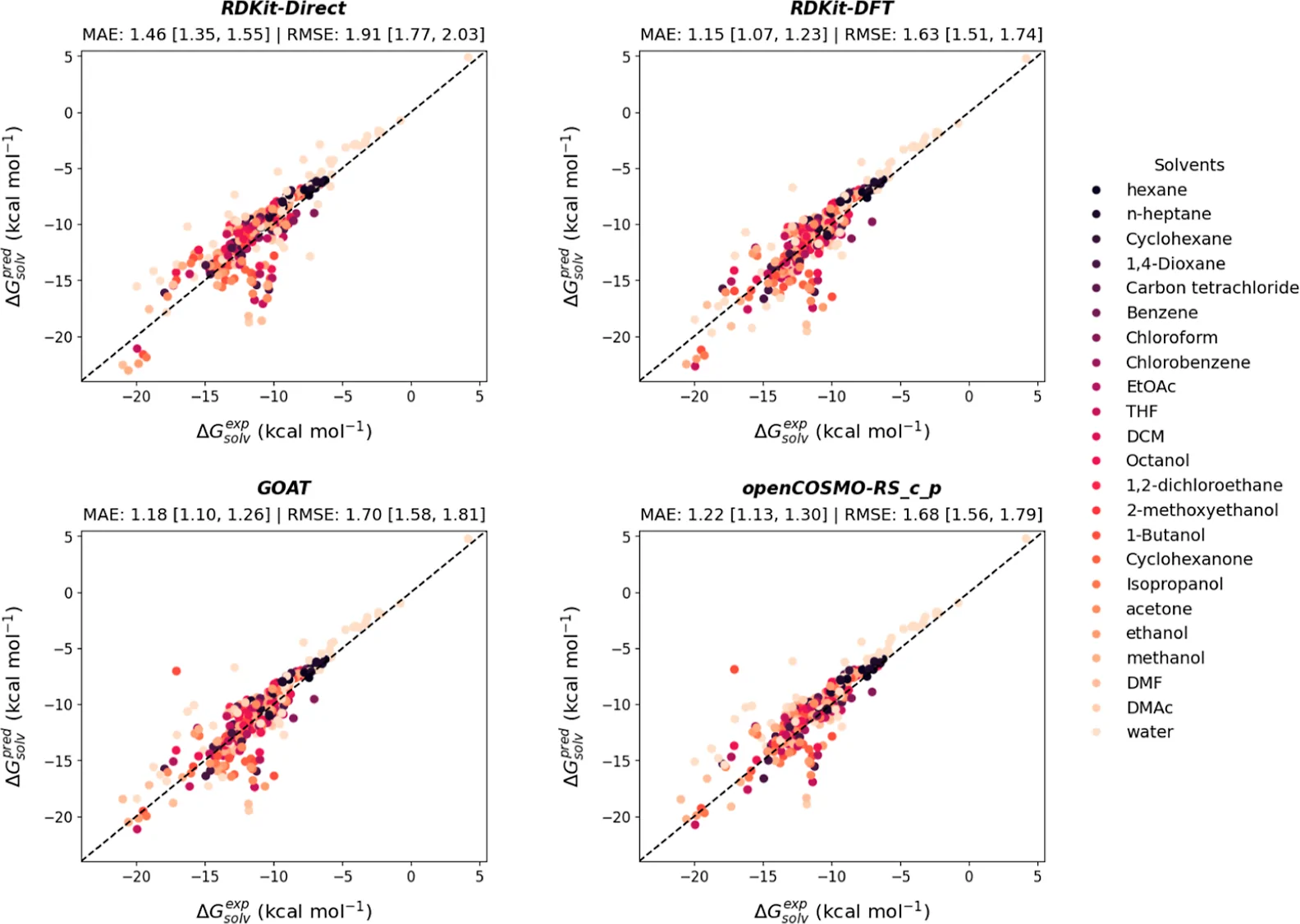
Parity plots comparing the predicted (Δ*G*_*solv*_^*pred*^) and experimental (Δ*G*_*solv*_^*exp*^) values, in kcal mol^–1^, for the different conformer generation workflows across the total Δ*G*_solv_ data set (*N* = 405 data points). Dashed black lines represent perfect parity (1:1) and are not fitted to the data. Data points are color-coded by a qualitative solvent polarity gradient, from dark (less polar) to light (more polar). The MAE and RMSE values are reported with the associated 95% confidence intervals. Pairwise statistical comparison of MAEs and RMSEs using correlated composite uncertainty is reported in Tables S7 and S8, respectively.

### COSMO-RS and Conformer-Generation Workflows: Are There Any Differences?

The above results show that, despite producing distinct input conformations, the studied workflows yield similar average performances for COSMO-RS calculations when the full data set is considered. While some subtle differences can be found for specific regimes (solubility and number of torsions), one might generally ask if choosing a specific workflow comes with any particular advantage, thus eventually helping in the definition of heuristic rules for molecule processing. In this context, one of the first issues one might consider is the computational efficiency. We performed a comparative analysis with the two representative molecules used earlier, paracetamol (ID 175 in the xlsx file, see Supporting Information) and glyburide (ID 174 in the xlsx file, see Supporting Information), by recording the total computational time of each workflow (Table 1). The four evaluated methods differ substantially in terms of methodological complexity and, hence, computational costs. For the smaller paracetamol, the timings are on the order of minutes; however, both ***openCOSMO***-***RS_c_p*** and ***GOAT*** workflows are 1 order of magnitude above ***RDKit***-***Direct***, which, besides being faster, provides a substantially lower deviation between calculated and experimental values (Figure 9). For the larger glyburide, ***openCOSMO***-***RS_c_p*** and ***GOAT*** require several hours, in contrast with the 5 min spent on the calculation of the solubility using the ***RDKit***-***Direct*** workflow. However, a larger accuracy is obtained using ***GOAT***, which is the best approach for this molecule. Curiously, the also computationally demanding ***openCOSMO*-*RS_c_p*** workflow yields the worst results. This indicates that the variations in molecular structures generated by the respective conformer generation methods do not substantially affect the average accuracy of solubility predictions for the full data set but definitely impact the results on specific molecules, as observed earlier.^16,23,24,27^

**Table 1:**
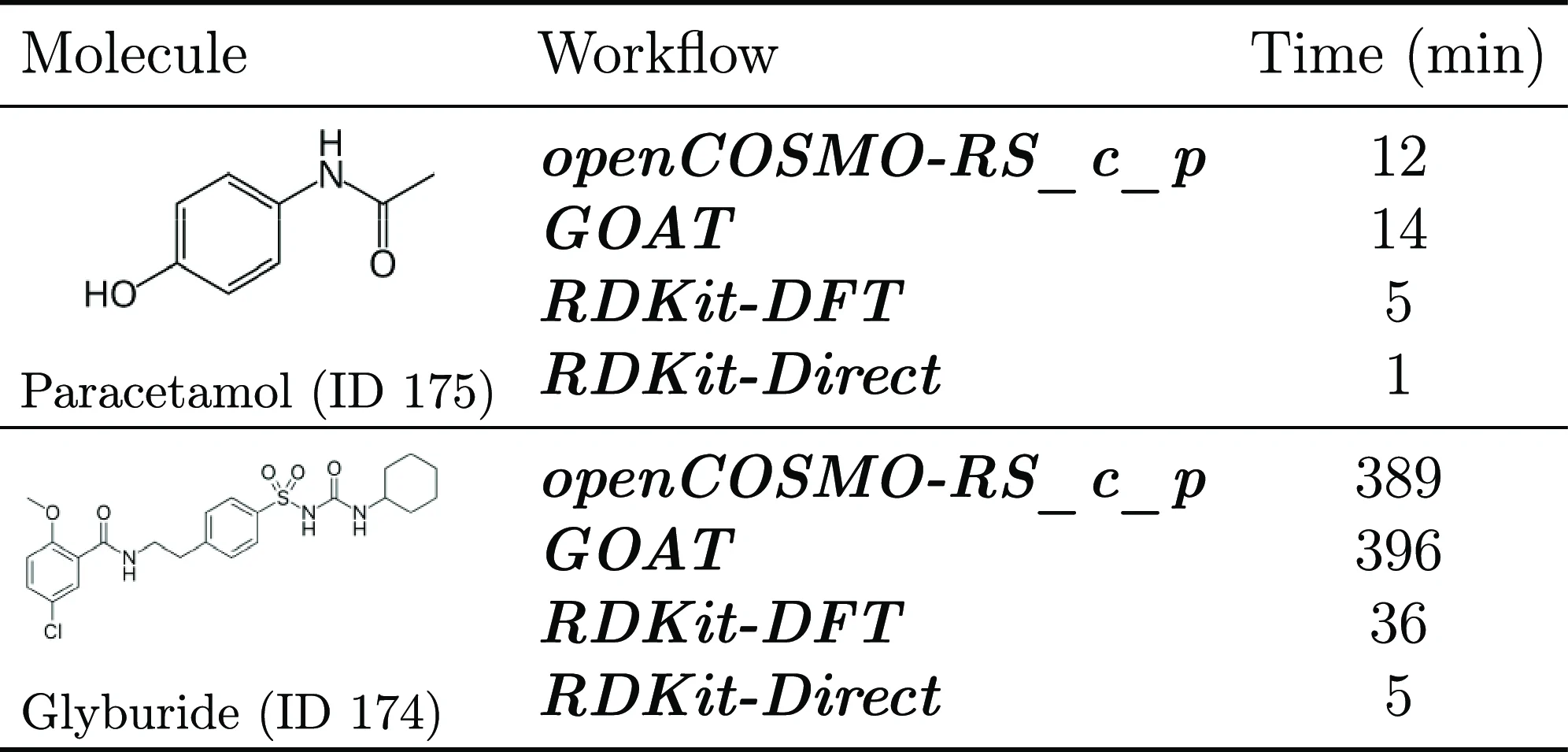
Total Computation Time (in Minutes) for the Conformer-Generation Workflows^a^

a All jobs were executed under identical conditions, namely, a single machine equipped with 8 CPU Intel­(R) Xeon­(R) Silver 4314 CPUs (2.40 GHz) and 64 GB of RAM.

**Figure 9:**
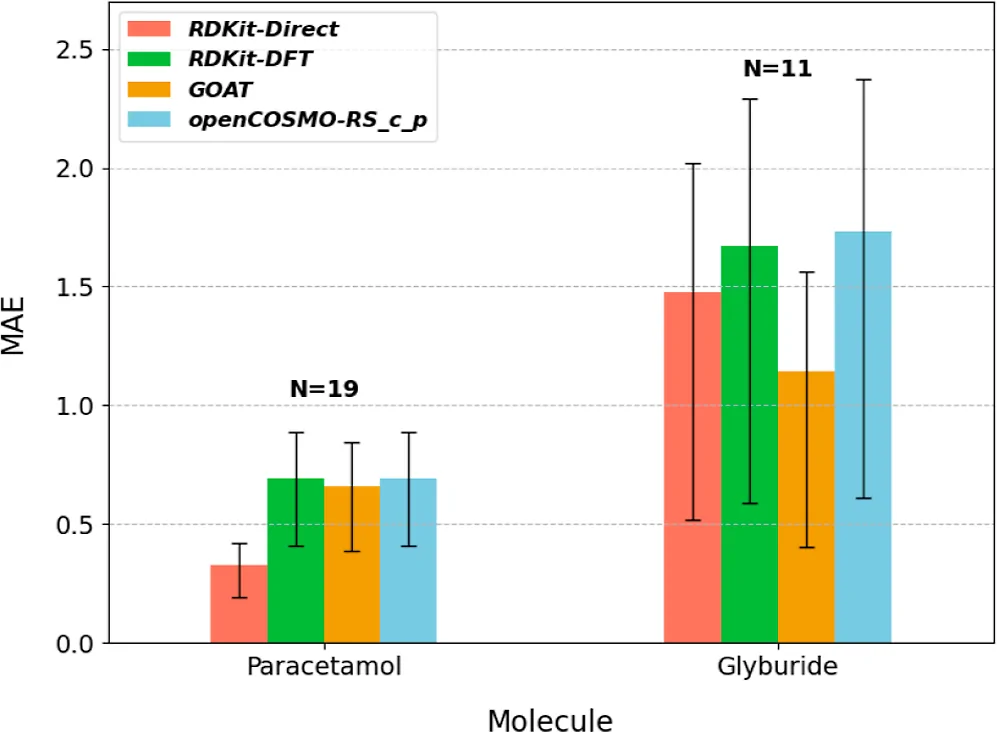
Comparison of the MAE values for paracetamol and glyburide using the different workflows along with the corresponding estimates of the 95% confidence intervals. The number of data points (*N*) used for the calculation of the MAE for each molecule is also shown.

To further assess this issue and explore potential avenues for improving automated pipelines for high-throughput screening, we investigated possible refinements to the conformer-generation step. Specifically, we considered the explicit incorporation of solvent-specific effects during the lowest-energy conformer search (single-conformer approach) and the usage of Boltzmann-weighted conformational ensembles, both implemented as variations of the ***GOAT*** pipeline. Regarding solvent effects, it is worth noting that, for all other workflows considered (except ***GOAT***), the initial conformations are generated using the ETKDG algorithm (or closely related variants), without providing any solvent-specific information at this stage of the conformer-generation process. In turn, throughout the conformational search performed by the GOAT method, the specific solvent environment can be explicitly taken into account on the GFN2-xTB potential energy surface. We therefore used the analytical linearized Poisson–Boltzmann (ALPB) solvation model to provide a solvent-specific lowest energy conformation, which was used to calculate the solubility. In addition to solvent-specific optimizations, Boltzmann-weighted conformer ensembles, generated using the GOAT algorithm, were also independently explored. The details of these calculations are given in Supporting Information Given the large number of data points and the computational cost of the specific ***GOAT*** pipeline, in these experiments, we focused on molecules where prediction errors were markedly pronounced or the molecular structure exhibited features suggestive of rich conformational diversity and/or a geometric sensitivity to the solvent. Such features include a relevant number of rotatable bonds, presence of hydrogen-bond donors and acceptors, or some degree of solute–solvent mismatch (e.g., an apolar molecule in a polar solvent). It should also be noted that certain solvents of interest (i.e., cyclohexane and *n*-heptane) were not available within the ALPB solvation model implemented in ORCA 6.0; thus, for these cases, the closest available alternatives were employed for the lowest-energy conformation generation step.

The results, summarized in Table 2, show that for most cases, the differences between the standard pipeline, (log_10_(*S*_calc_^std^)), and modifications considering the solvent or the use of a conformer ensemble, (log_10_(*S*_calc_^opt^)) and (log_10_(*S*_calc_^ens^)), respectively, are less than 5% (uncolored cells). However, when changes do occur, the agreement with the experiment improves (green cells), while worsening is found only for paracetamol in cyclohexane using an ensemble. We focus now on the values obtained with a solvent-specific modification of the ***GOAT*** pipeline (log_10_(*S*_calc_^opt^)). The use of the solvent information in the lowest-energy conformer search often did not change the results, as already mentioned above. This observation is concomitant with the fact that the differences between the default geometry and the solvent-dependent one are negligible for some compounds, namely, paracetamol in all solvents (Figure S10) or omeprazole in all solvents except ethanol (Figure S11). However, the magnitude of the RMSD by itself is not a good descriptor of the geometry influence on the calculated solubility, as musk xylene or glyburide presents similar $\log_{10}\left(S_{calc}^{std}\right)$ and $\log_{10}\left(S_{calc}^{opt}\right)$ values despite the non-negligible RMSD values. In these compounds, the solvent-dependent structures differ primarily in the rotation of groups without a substantial change in the exposed volume or the breaking/formation of intramolecular hydrogen bonds (see Figures S12 and S13 for musk xylene and glyburide, respectively). Nonetheless, positive changes are observed for cimetidine (all solvents), sorbitol, and omeprazole (in both ethanol). If one analyzes the solvent-dependent low-energy conformations obtained for cimetidine (Figure 10), where a large accuracy of log_10_(S_calc_^opt^) is obtained, a noticeable change is observed: the presence of a hydrogen bond that was absent on the standard ***GOAT*** pipeline. The presence of this hydrogen bond appears to be responsible for a better description of the molecule, leading to a better COSMO-RS solubility prediction. These changes in the hydrogen-bonding patterns were also observed for sorbitol (Figure S14) and omeprazole (Figure S11) in ethanol, leading to improved accuracy. The results are in line with those previously reported for vanillin and ethyl vanillin, where the rotation of a hydroxyl group and the formation of an intramolecular hydrogen bond significantly impacted the accuracy of COSMO-RS solubility predictions.^16^

**Table 2:**
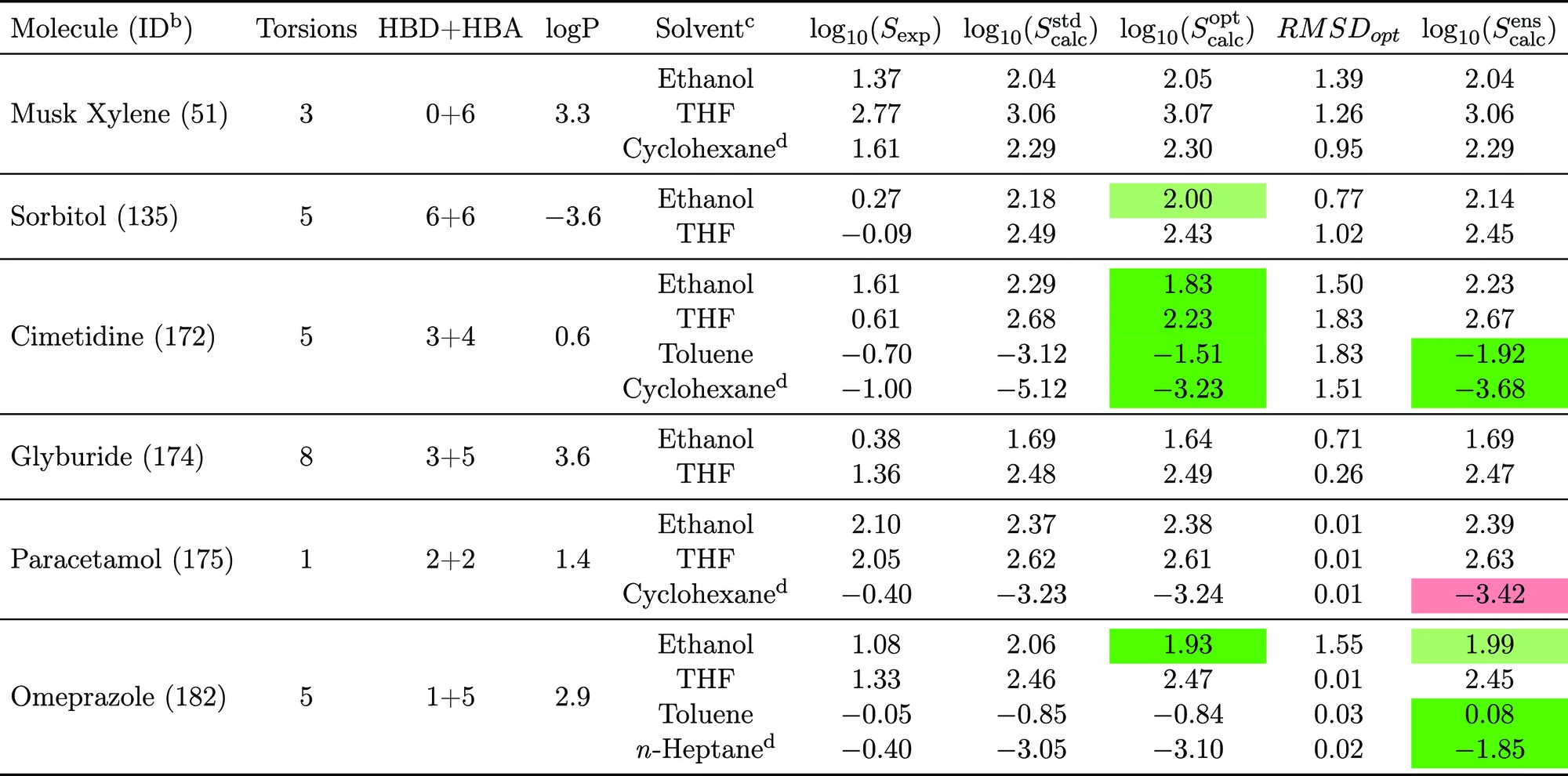
Experimental and Calculated Solubilities (log_10_(*S*), Where *S* Is in mg mL^–1^) for Selected Compounds Using the ***GOAT*** Pipeline^a^

a The log_10_(*S*_calc_^std^) refers to values calculated with the standard pipeline, whereas log_10_(*S*_calc_^opt^) and log_10_(*S*_calc_^ens^) values were obtained using the solvent-specific modification and Boltzmann–weighted conformers, respectively. Selected molecular descriptors are shown along with the corresponding root-mean-square deviations of the atomic coordinates (in Å) between the structures used for *S*_calc_^std^ and *S*_calc_^opt^ (RMSD_opt_). Cells are colored only when log_10_(*S*_calc_^std^) and log_10_(*S*_calc_^opt^)/log_10_(*S*_calc_^ens^) differ by more than 5%; light green indicates moderate improvement (5–10% closer to the experiment), dark green indicates strong improvement (≥10% closer to the experiment), and red indicates worsening (≥5% farther from the experiment).

b IDs correspond to the Supporting Information xlsx file.

c The dielectric constants are as follows: ethanol (24.5), THF (7.6), toluene (2.4), cyclohexane (2.0), and *n*-heptane (1.9).

d Optimized in the closest available alternative: hexane (dielectric 1.9).

**Figure 10:**
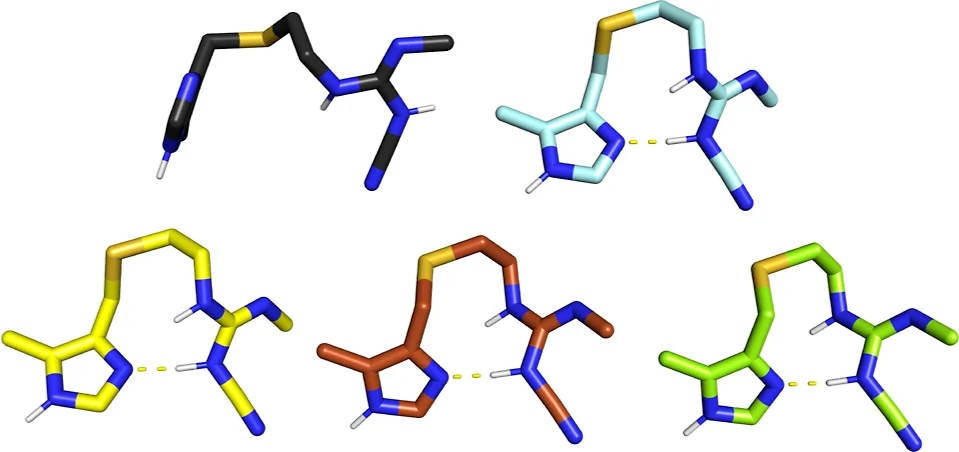
Solvent-dependent low-energy conformations obtained for cimetidine (ID: 172) using the ***GOAT*** pipeline: standard (gray), ethanol (yellow), THF (brown), cyclohexane (cyan), and toluene (lemon green). The nonpolar hydrogen atoms were omitted for clarity. The hydrogen bonds are represented as yellow dashes.

When employing Boltzmann-weighted conformer ensembles to assess solubility (log_10_(*S*_calc_^ens^) values in Table 2), we observe improvements similar to those obtained with the solvent-specific optimization strategy; however, the magnitude of the enhancement is generally less pronounced. In the case of omeprazole, improvements are also observed in toluene and *n*-heptane, presumably because of the presence of multiple energetically accessible conformations that differently expose polar and nonpolar groups to the solvent environment. Nevertheless, this behavior does not appear to be a general trend. For example, for a relatively simple molecule such as paracetamol in cyclohexane, the use of a conformational ensemble led to a deterioration of the prediction. These observations suggest that if obtained using a solvent-specific search, a single conformation often suffices in capturing the dominant contributions, yielding results that are reasonably accurate and competitive with Boltzmann-weighted ensembles while offering a more favorable trade-off in terms of computational cost. It is also important to emphasize that these findings were obtained within the constraints and limits of the openCOSMO-RS 24a parametrization, and no attempt was made for further optimization. Consequently, even with an optimal molecular geometry, the achievable accuracy is ultimately limited by the intrinsic performance of this parameter set, which was developed for a different chemical space (Figure S1). Newer parametrizations of the openCOSMO-RS model are now available for halocarbons^62^ and to account for atomic polarizabilities,^63^ and their application may further refine quantitative agreement with experiment, particularly in challenging solubility domains.

## Conclusions

In this work, we systematically evaluated the influence of conformer generation strategies for COSMO-RS solubility and Gibbs free energy of solvation predictions using a large and chemically diverse data set. As the accuracy of these predictions can strongly depend on the input geometry, this evaluation provides timely and practical guidance for defining computational workflows for the solubility prediction.

Four representative conformer generation methods, ranging from simple RDKit-based distance-geometry approaches to more elaborate semiempirical (GFN2-xTB/GOAT) and DFT-based pipelines, were assessed. Despite the methodological differences in complexity and computational cost, leading to distinct conformations, all workflows yield similar average performances when the full data set of 926 data points is considered. While subtle differences emerge in specific regimes, particularly for poorly soluble compounds and more flexible molecules, no workflow consistently outperforms the others across all conditions. The capability of the workflows to rank compounds, as measured by Spearman’s rank correlation coefficient, was also limited.

For high-throughput screening purposes, the selection of a conformer generation and modeling approach should be guided not only by considerations of accuracy but also by computational efficiency and the intended use. In this context, the fast and simple ***RDKit***-***Direct*** might be a justified and effective solution. Indeed, the predominance of relatively rigid molecules in our data set would be expected to yield similar performance across workflows rather than systematically favoring the simplest approach. This observation indicates that the differences cannot be explained solely by molecular flexibility and instead point to the influence of additional factors contributing to the observed performance disparities.

However, the results also showed that for molecules with substantial conformational flexibility, particularly those with more than five rotatable bonds and the ability to form intramolecular hydrogen bonds, the choice of the conformer generation strategy becomes a decisive factor. Therefore, targeted refinements of the conformer generation step were also explored. Incorporating solvent-specific effects during the conformational search or employing Boltzmann-weighted conformer ensembles can improve predictions in selected cases, particularly when intramolecular hydrogen bonding or solvent-dependent structural rearrangements are involved. In several situations, a solvent-specific single conformation provides results comparable to those obtained from the conformational ensembles. Nonetheless, we point out that methods that offer users the ability to select such possibilities (solvent-specific potential energy surface exploration or ensemble generation), such as ***GOAT***, seem to be a well-suited platform for achieving reliable molecular representations in these more conformationally complex and challenging systems. We also point out that for obtaining solvent-aware conformations, recent machine-learning approaches, such as the graph neural network-based implicit solvent model,^64^ are also promising and illustrate ongoing efforts toward integrating data-driven models with traditional computational chemistry workflows to better capture solvation-induced structural variability.

Overall, the results indicate that, within the limits of the openCOSMO-RS 24a parametrization, the choice of the conformer-generation workflow has a moderate impact on average predictive accuracy for large data sets such as the one employed here, although it can significantly influence specific molecules or particularly challenging regimes. Future improvements in COSMO-RS parametrizations, together with a careful selection of conformer-generation strategies, may further enhance quantitative agreement, especially for poorly soluble compounds and flexible systems. The findings reported here may also offer useful guidance for the development of heuristic rules for the automated molecular screening.

## Supplementary Material





## Data Availability

Experimental data for the solubility were taken from BigSolDB 2.0, available in https://bigsoldb.cheminfo.space/) and refs 11,41 . Experimental values of free energies of solvation are available in GitHub (https://github.com/BioE-KimLab/Solv_GNN_SSD). A curated version of the data used in this work is provided as an xlsx file in the Supporting Information. All QM calculations were performed at BioISI with ORCA, version 6.0, which can be obtained free of charge for academic and personal use (https://www.faccts.de/orca/). For the ***openCOSMO***-***RS_c_p*** workflow, the openCOSMO-RS conformer pipeline, available at https://github.com/TUHH-TVT/openCOSMO-RS_conformer_pipeline, was employed. COSMO-RS calculations were performed using the openCOSMO-RS 24a Python implementation freely available in https://github.com/TUHH-TVT/openCOSMO-RS_py.

## Notes

Principal component analysis of molecular fingerprints comparing our solubility data set and the openCOSMO-RS 24a; distribution of the prediction errors for the different conformer generation workflows; RMSE values for each solvent; cumulative RMSE calculated over increasing subsets of experimental solubility; cumulative Spearman’s rank correlation coefficient; MAE and RMSE of the Δ*G*_solv_ for each solvent; solvent-dependent lowest energy conformations for Paracetamol, Omeprazole, Musk Xylene, Glyburide, and Sorbitol; and details of the Boltzmann–weighted conformer ensemble calculations (PDF)
Data set of 926 data points, containing the experimental and calculated solubility values of 185 molecules across 36 solvents (XLSX)
