# Supplementary material for: Conformer Generation Workflows for COSMO-RS Calculations: Are They All the Same?
Source: J Chem Inf Model. 2026 May 14;66(16):10004–18. doi: 10.1021/acs.jcim.6c00606 (PMC13508778; doi:10.1021/acs.jcim.6c00606)
Supplement: Supplementary file 1 [file ci6c00606_si_001.pdf]

Supporting Information: Conformer Generation Workflows for COSMO-RS Calculations: Are They All the Same?

André M. M. Gomes,†,‡ José F. O. Granjo,*,‡ and Paulo J. Costa*,†

†*BioISI – Instituto de Biossistemas e Ciências Integrativas, Faculdade de Ciências, Universidade de Lisboa, 1749-016, Lisbon, Portugal and Departamento de Química e Bioquímica, Faculdade de Ciências, Universidade de Lisboa, Lisbon, Portugal*

‡*Hovione R&D, Hovione FarmaCiência S.A., Estrada do Paço do Lumiar, 1649-038 Lisbon, Portugal*

E-mail: jogranjo@hovione.com; pjcosta@ciencias.ulisboa.pt

The Supporting Information includes

Supporting Figures **S-3**

Supporting Tables **S-11**

Boltzmann–Weighted Conformer Ensemble Calculations **S-14**

References **S-16**

Supporting Figures

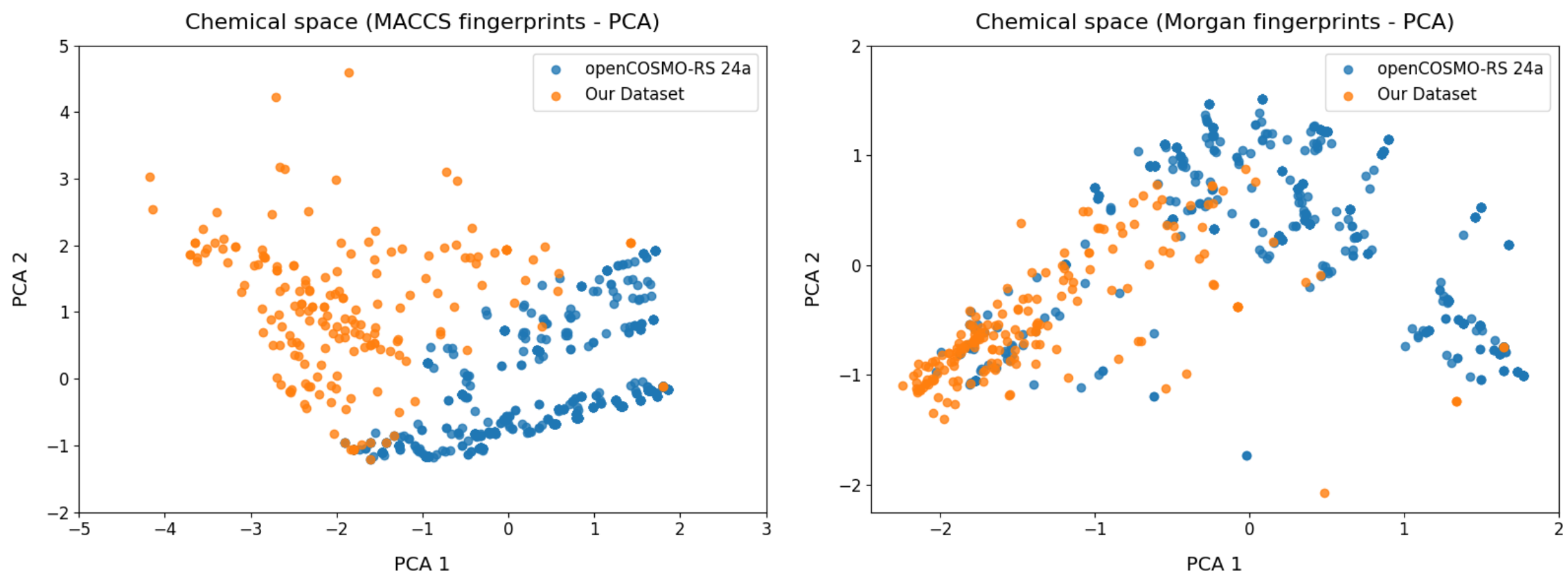

Figure S1: Principal component analysis (PCA) of molecular fingerprints comparing our solubility dataset and the openCOSMO-RS 24a training set:[S1] (left) MACCS fingerprints; (right) Morgan fingerprints. Both datasets are projected onto the first two principal components to assess domain similarity in chemical space.

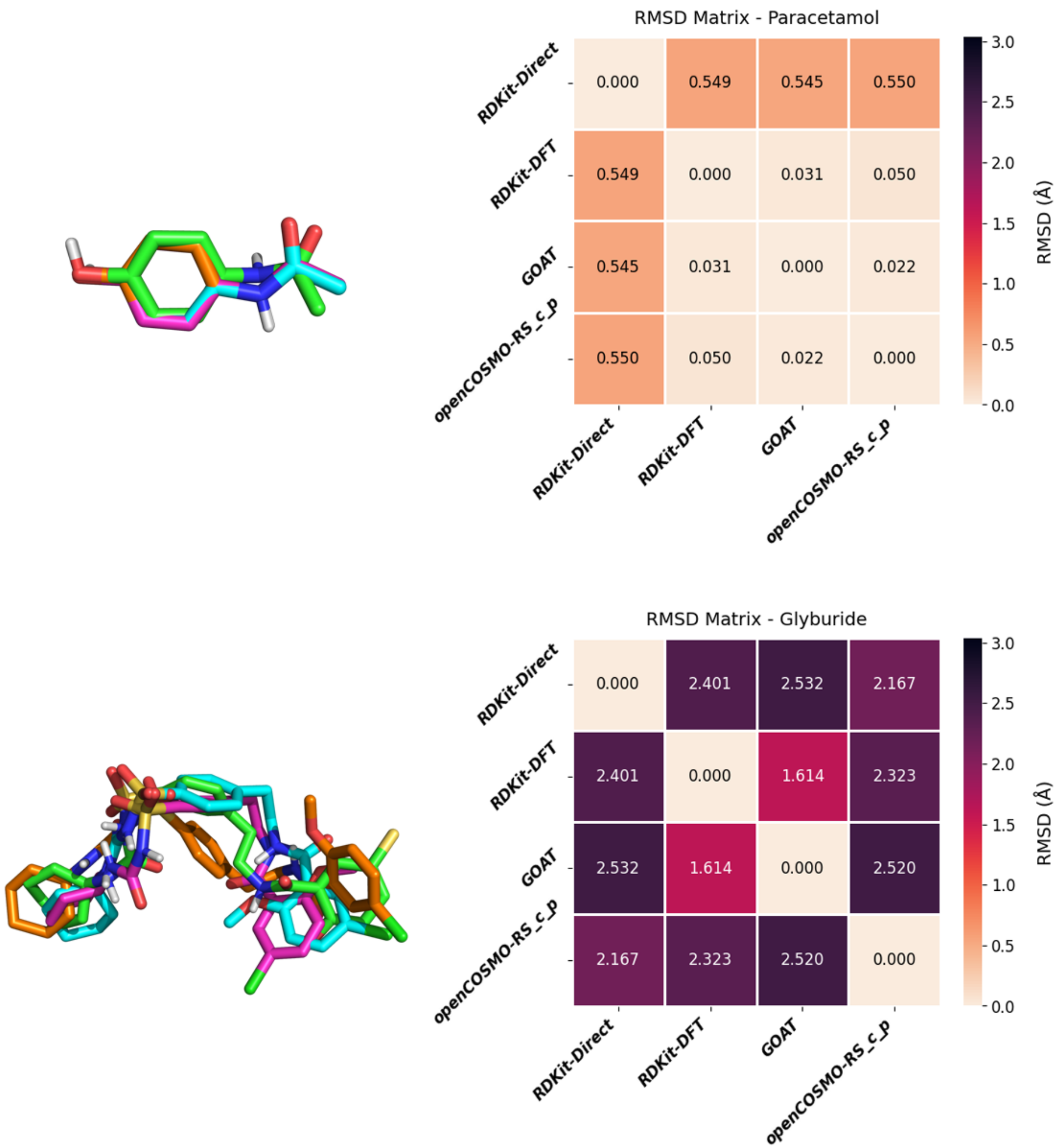

Figure S2: Left: Overlay of the four aligned final structures of paracetamol (top panel) and glyburide (bottom panel) generated using studied workflows: ***RDKit-Direct*** (green), ***RDKit-DFT*** (blue), ***GOAT*** (pink), and ***openCOSMO-RS_c_p*** (orange). Right: Matrix of the mean cross RMSD values (in Å) between atomic coordinates of the final conformers.

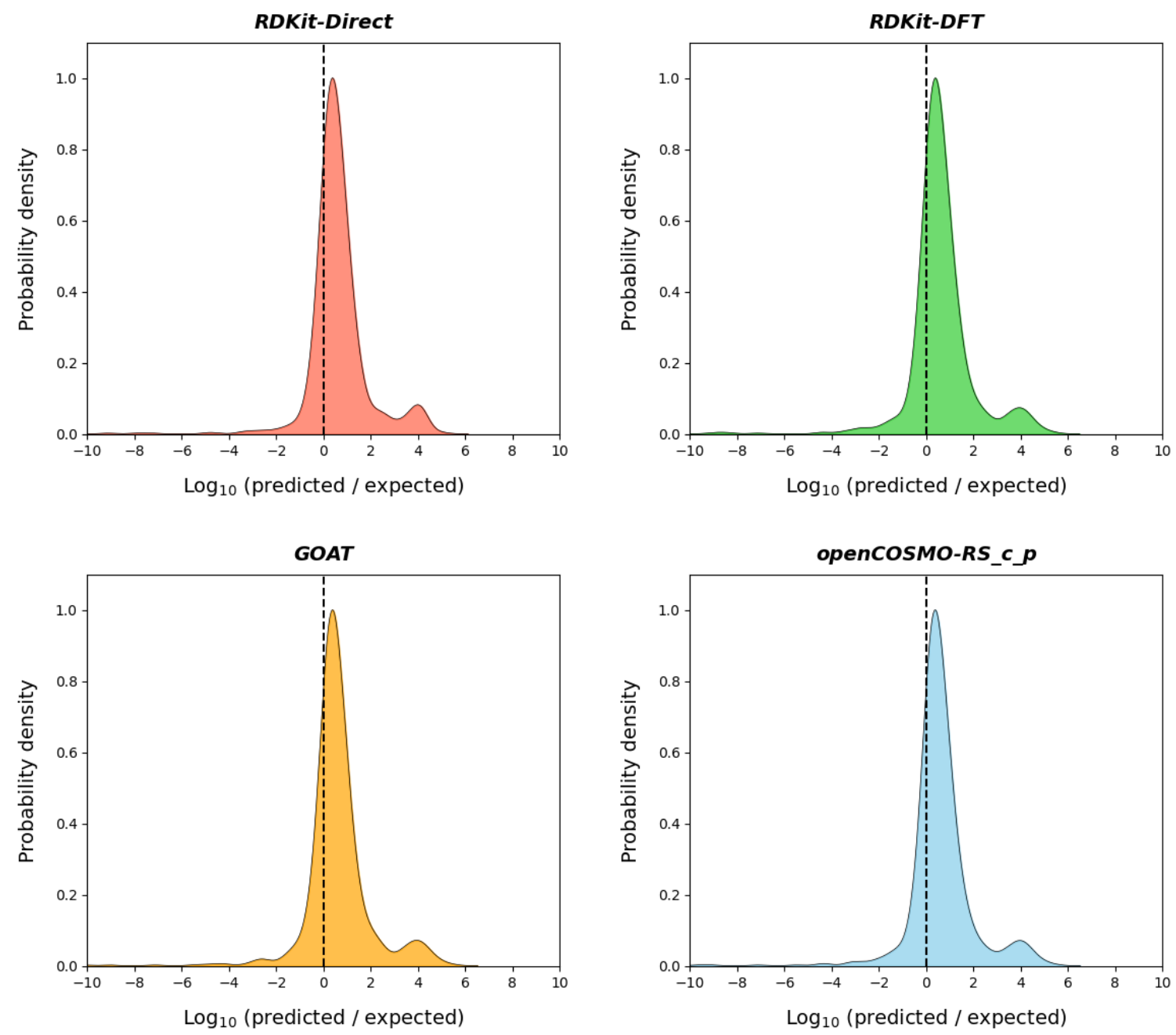

Figure S3: Distribution of the prediction errors for the different conformer generation workflows (data in logarithmic scale, base 10). Dashed black lines represent parity (1:1) and are not fitted to the data.

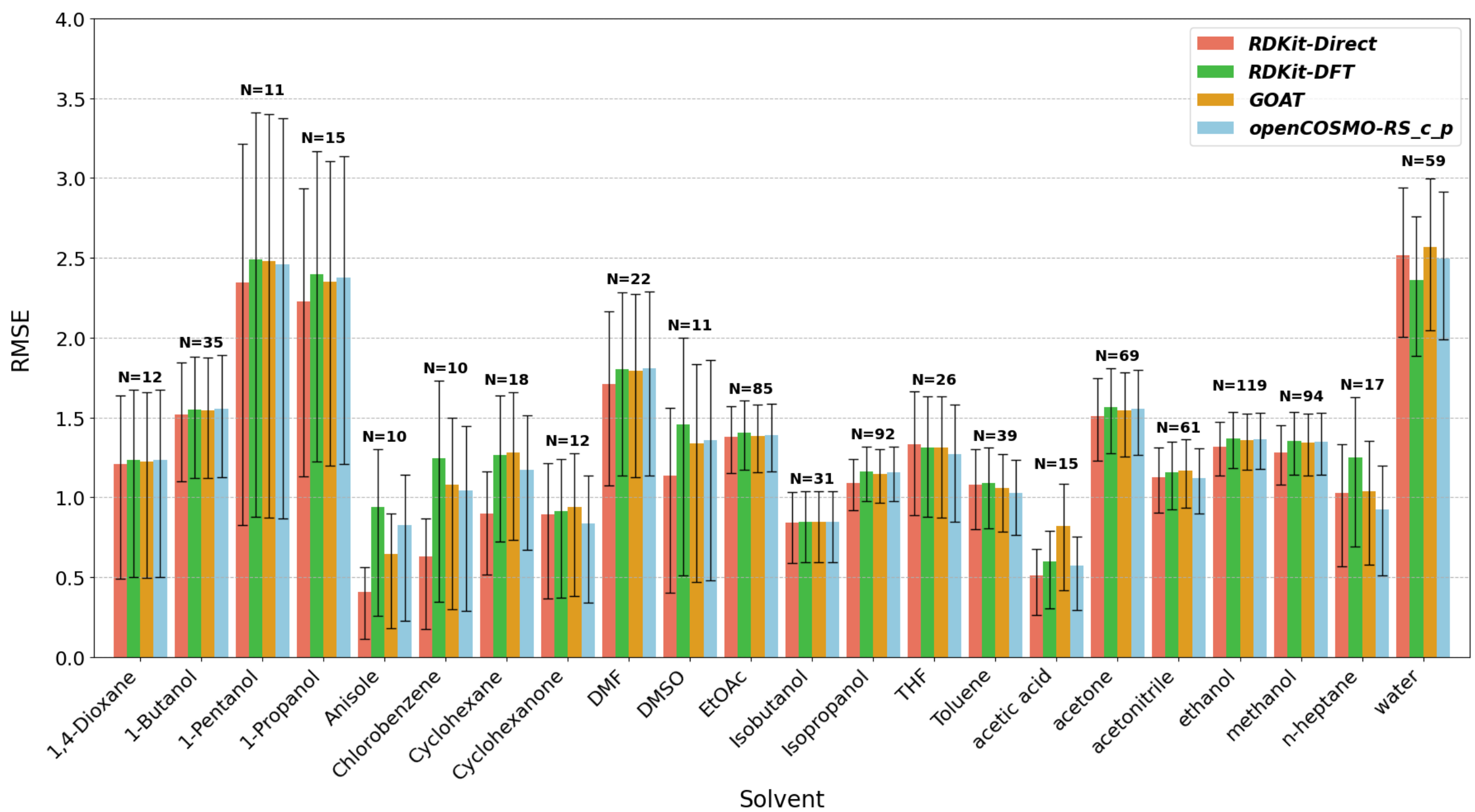

Figure S4: RMSE for solubility (in $\log_{10}$ mg mL^{-1}) between calculated and experimental values for each solvent and each conformer generation workflow. The number of data points (N) used for the calculation of the RMSE for each solvent is also shown. Only solvents with $N > 8$ were included in the plot.

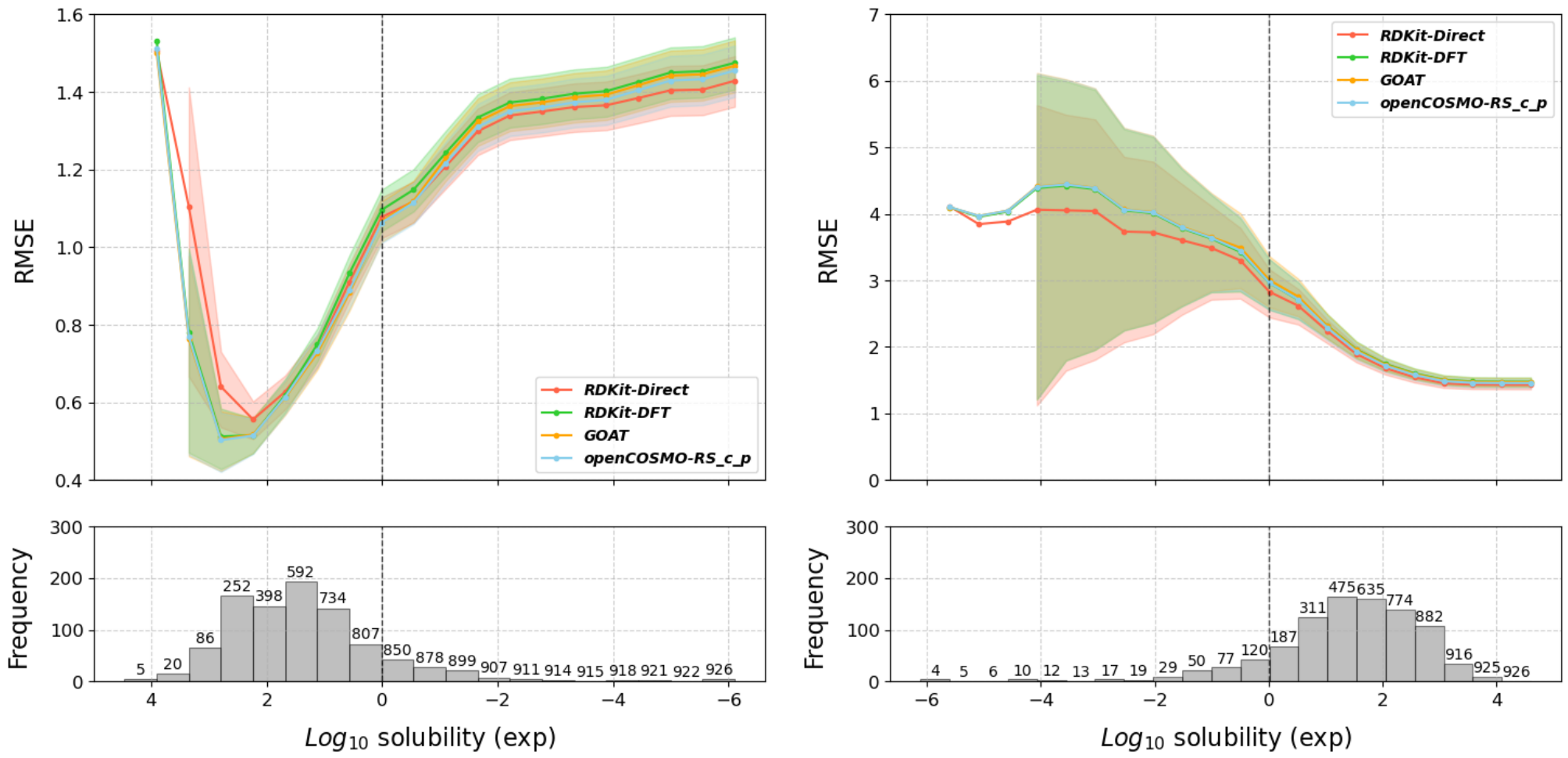

Figure S5: Cumulative RMSE (in $\log_{10}$ mg mL^{-1}) for each conformer-generation pipeline, calculated over progressively larger subsets of experimental solubility data. Subsets are ordered from high to low (left panels) or from low to high (right panels) solubility. Shaded areas represent the associated 95 % confidence intervals (for cases when the number of data points $N > 8$). The bottom panels display the bin populations together with the cumulative N used in each successive RMSE calculation. A uniform bin width of 0.5 log units was employed. The dashed vertical lines indicate the 1 mg mL^{-1} solubility threshold.

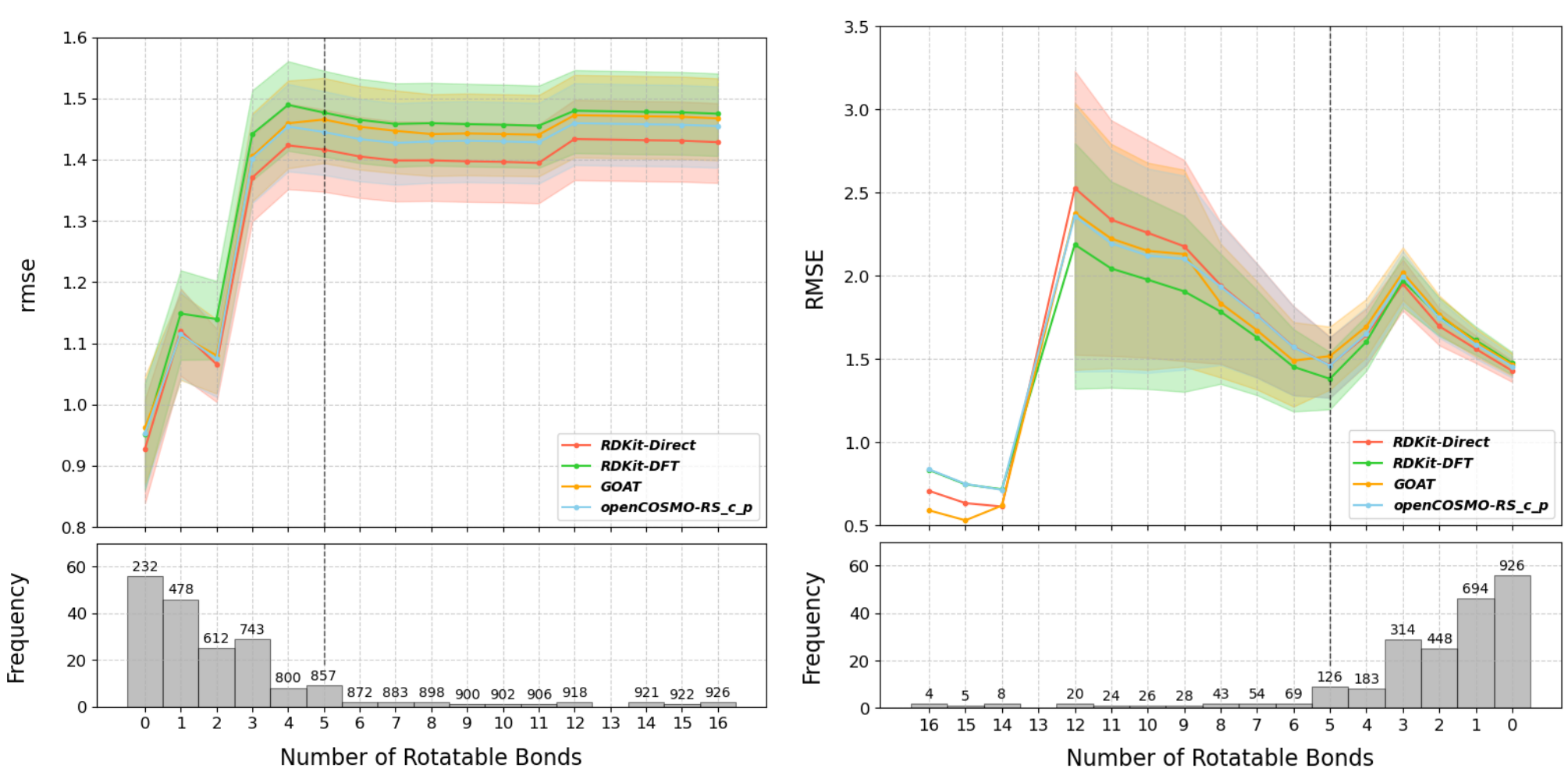

Figure S6: Cumulative RMSE (in $\log_{10}$ mg mL^{-1}) for each conformer-generation pipeline, computed over progressively larger subsets of molecules ordered by the number of rotatable bonds, from high to low (left) and from low to high (right). Shaded areas represent the associated 95 % confidence intervals (for cases when the number of data points $N > 8$). The bottom panels display the bin populations together with the cumulative N used in each successive RMSE calculation. A uniform bin width of 0.5 log units was employed. The dashed vertical lines indicate a 5 rotatable bond threshold.

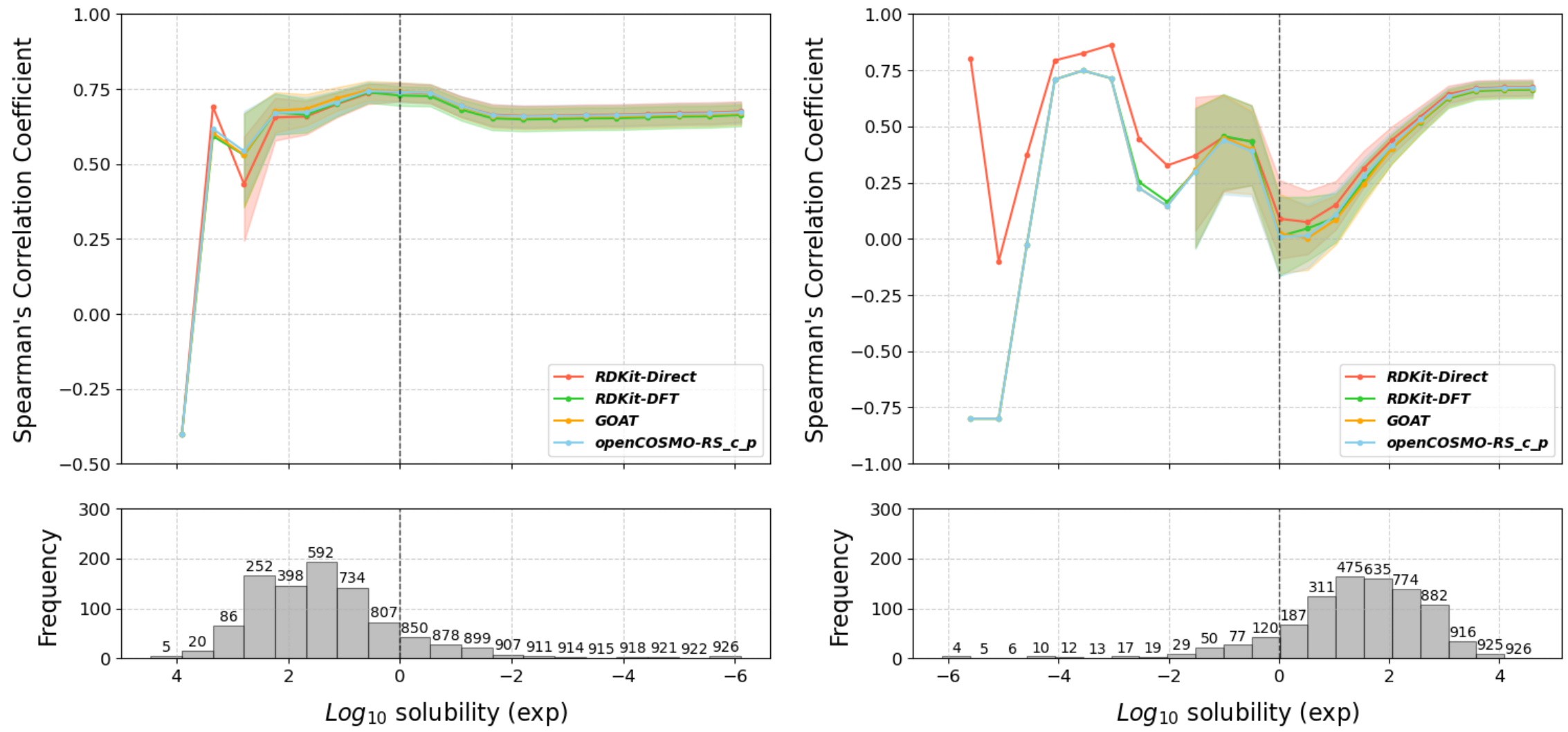

Figure S7: Cumulative Spearman's rank correlation coefficient for each conformer-generation pipeline, calculated over progressively larger subsets of experimental solubility data. Subsets are ordered from high to low (left panels) or from low to high (right panels) solubility. Shaded areas represent the associated 95 % confidence intervals (for cases when $N \geq 25$) estimated using a Fisher z-transformation. The bottom panels display the bin populations together with the cumulative number of data points (N) used in each successive calculation. A uniform bin width of 0.5 log units was employed. The dashed vertical lines indicate the 1 mg mL^{-1} solubility threshold.

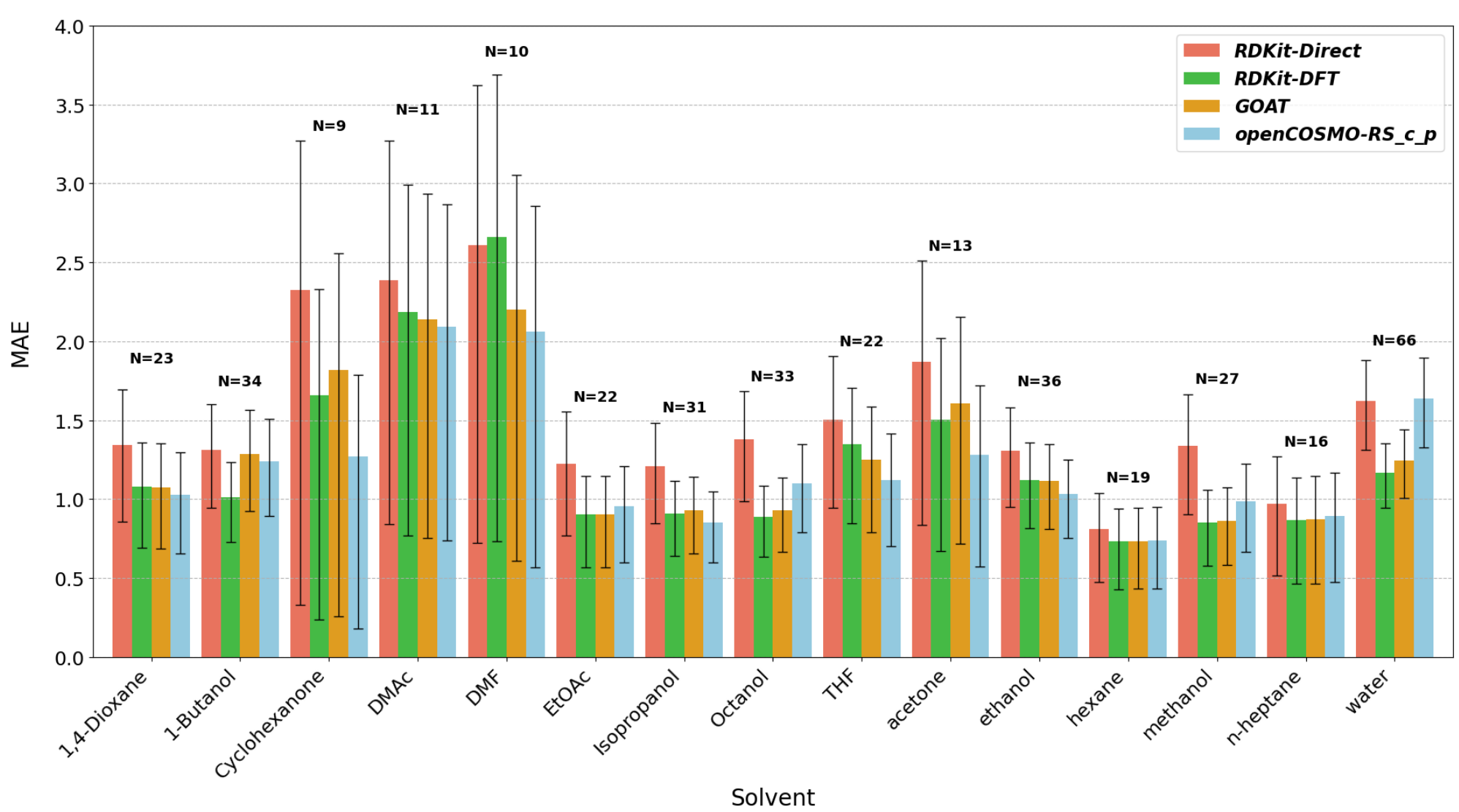

Figure S8: MAE between calculated and experimental ΔG_{solv} values (in kcal mol^{-1}) for each solvent and each conformer workflow. The number of data points (N) for each solvent is also shown. Only solvents with N > 8 were included in the plot. A similar analysis using the RMSE instead is shown in Figure S9.

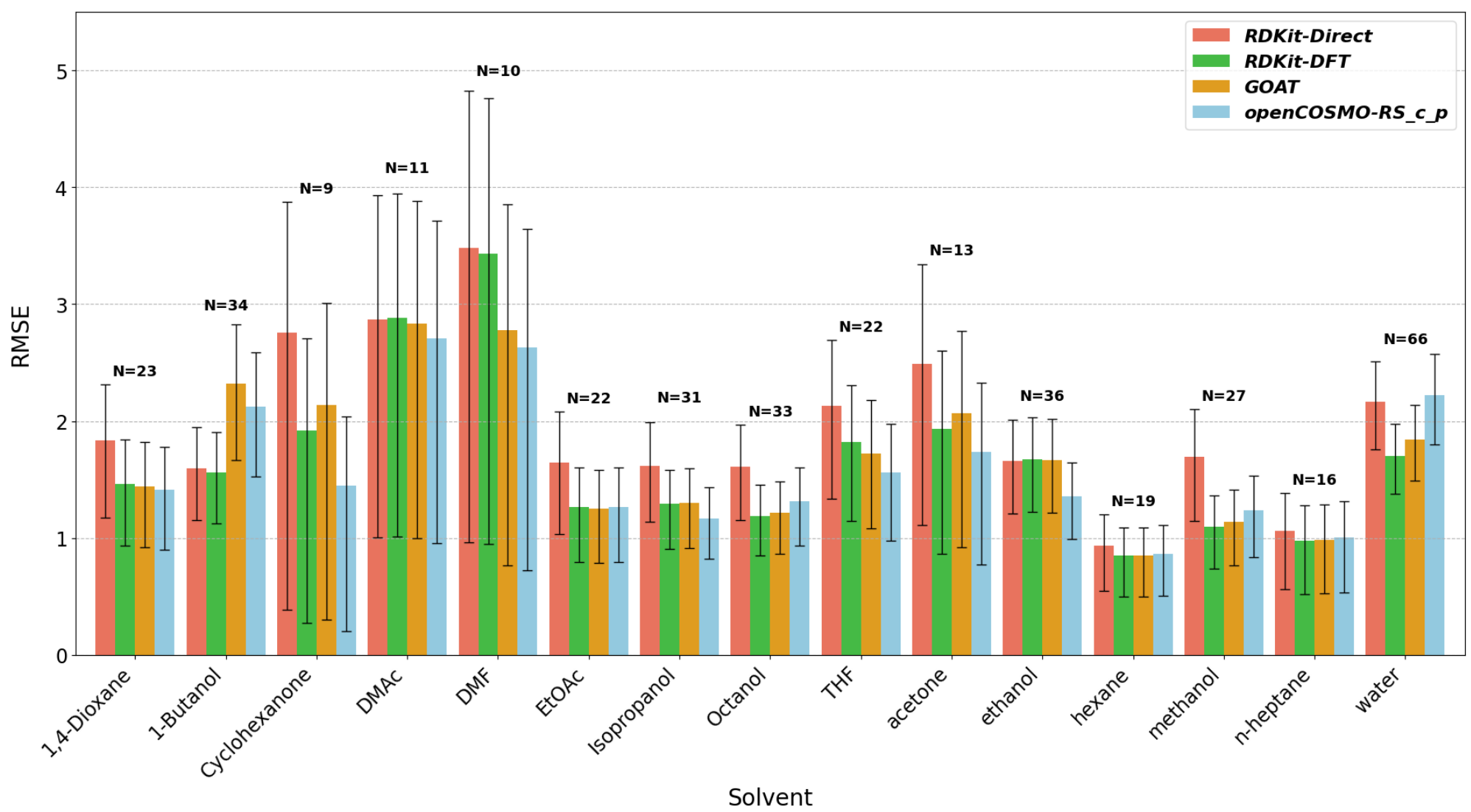

Figure S9: RMSE between calculated and experimental ΔG_{solv} values (in kcal mol^{-1}) for each solvent and each conformer workflow. The number of data points (N) for each solvent is also shown. Only solvents with $N > 8$ were included in the plot.

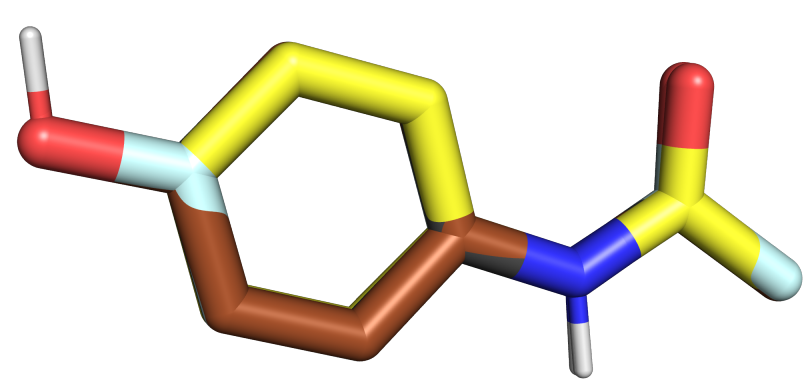

Figure S10: Superposition of the low-energy conformations obtained for paracetamol (175) using the ***GOAT*** pipeline: standard (gray), ethanol (yellow), THF (brown), and cyclohexane (cyan)

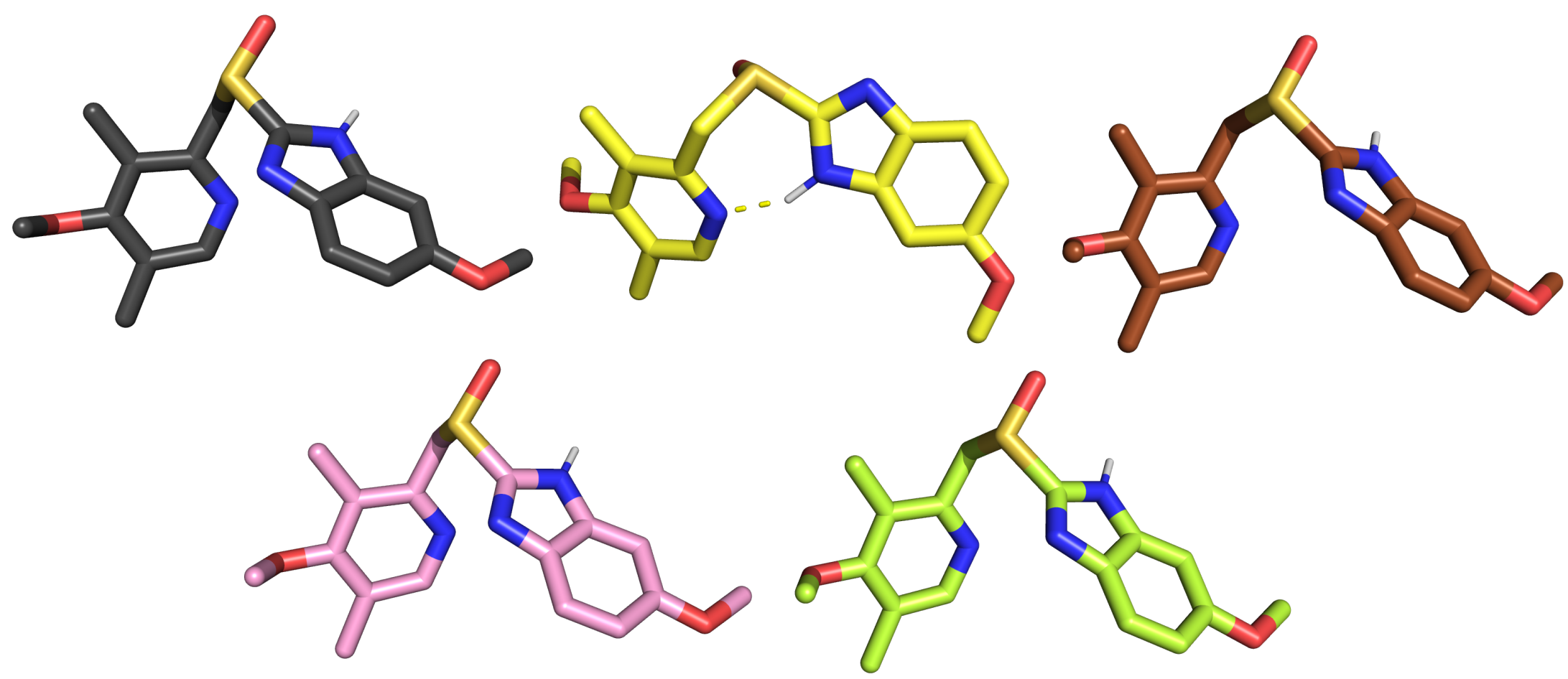

Figure S11: Superposition of the low-energy conformations obtained for omeprazole (182) using the ***GOAT*** pipeline: standard (gray), ethanol (yellow), n-heptane (pink), THF (brown), and toluene (lime green)

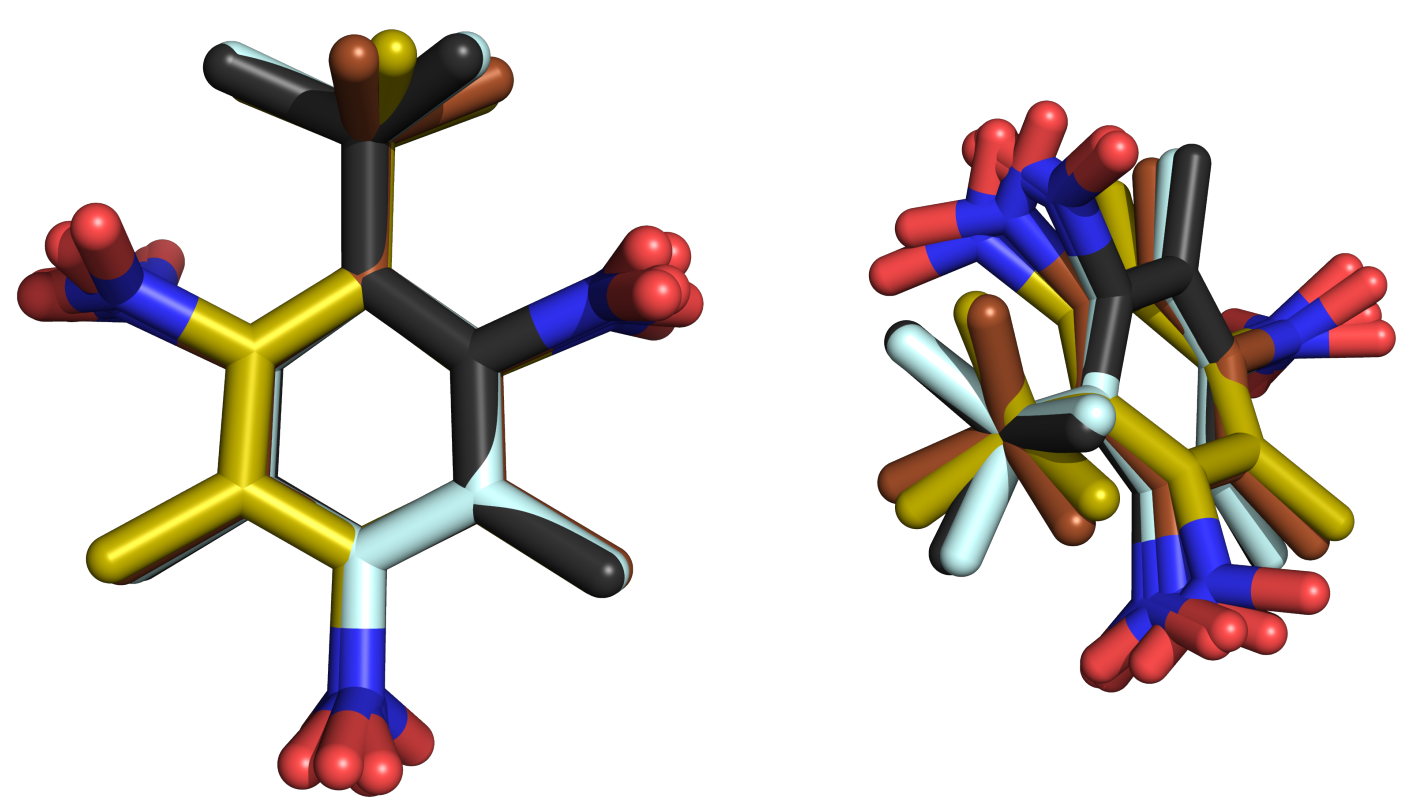

Figure S12: Superposition of the solvent-dependent low-energy conformations obtained for musk xylene (51) using the ***GOAT*** pipeline: standard (gray), ethanol (yellow), THF (brown), and cyclohexane (cyan).

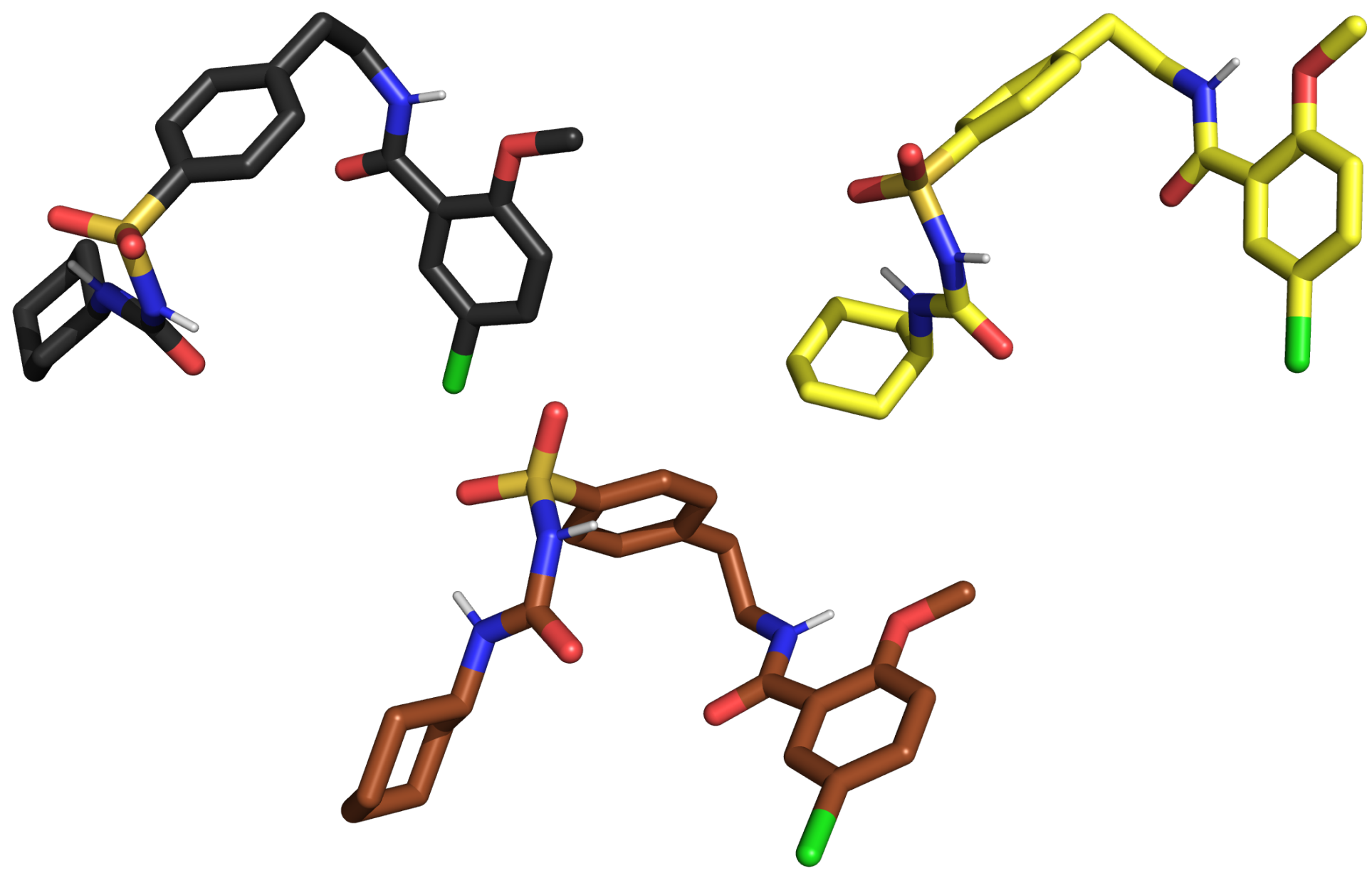

Figure S13: Low-energy conformations obtained for glyburide (174) using the ***GOAT*** pipeline: standard (gray), ethanol (yellow), and THF (brown).

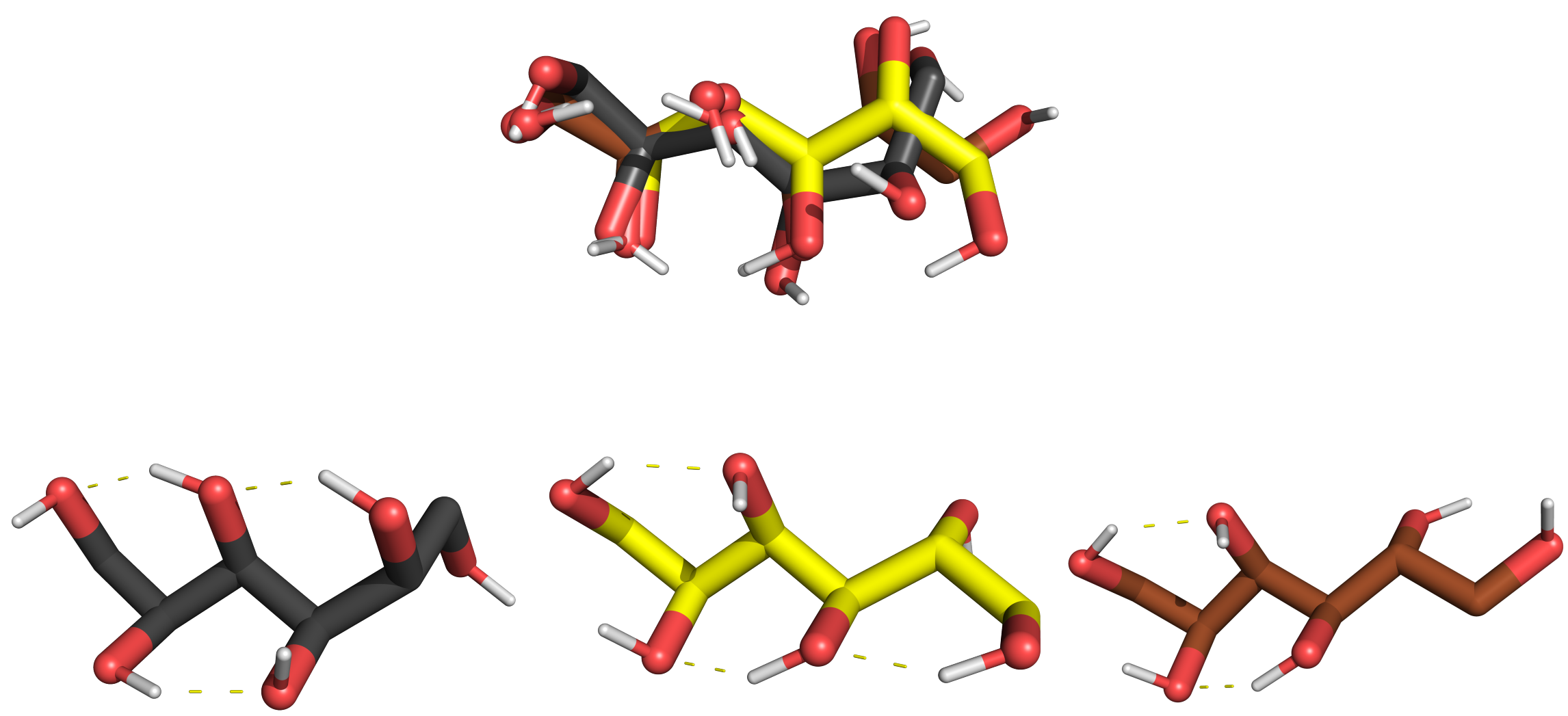

Figure S14: (Top) superposition of the solvent-dependent low-energy conformations obtained for sorbitol (135) using the ***GOAT*** pipeline: standard (gray), ethanol (yellow), and THF (brown); (Bottom) the hydrogen bonding pattern within the three individual structures is shown.

Supporting Tables

Table S1: Pairwise statistical comparison of the MAEs reported in Figure 4, obtained with the full dataset ($N = 926$) using the correlated composite uncertainty at the 95% confidence level. Each table entry indicates whether the difference in MAE between two methods is statistically significant (S) or not significant (NS), according to the comparison criterion (Equation 7).

	RDKit-Direct	***RDKit-DFT***	***GOAT***	***openCOSMO-RS_c_p***
RDKit-Direct	—	S	S	S
RDKit-DFT	S	—	S	S
GOAT	S	S	—	NS
openCOSMO-RS_c_p	S	S	NS	—

Table S2: Pairwise statistical comparison of the RMSEs reported in Figure 4, obtained with the full dataset ($N = 926$) using the correlated composite uncertainty at the 95% confidence level. Each table entry indicates whether the difference in RMSE between two methods is statistically significant (S) or not significant (NS), according to the comparison criterion (Equation 7).

	RDKit-Direct	***RDKit-DFT***	***GOAT***	***openCOSMO-RS_c_p***
RDKit-Direct	—	S	S	S
RDKit-DFT	S	—	NS	S
GOAT	S	NS	—	NS
openCOSMO-RS_c_p	S	S	NS	—

Table S3: Pairwise statistical comparison of the MAEs reported in Figure 6, left, obtained for points whose solubility is above 1 mg mL^{-1} ($N = 807$) using the correlated composite uncertainty at the 95% confidence level. Each table entry indicates whether the difference in MAE between two methods is statistically significant (S) or not significant (NS), according to the comparison criterion (Equation 7).

	RDKit-Direct	***RDKit-DFT***	***GOAT***	***openCOSMO-RS_c_p***
RDKit-Direct	—	S	NS	S
RDKit-DFT	S	—	S	S
GOAT	NS	S	—	NS
openCOSMO-RS_c_p	S	S	NS	—

Table S4: Pairwise statistical comparison of the MAEs reported in Figure 6, right, obtained for points whose solubility is below 1 mg mL^{-1} ($N = 120$) using the correlated composite uncertainty at the 95% confidence level. Each table entry indicates whether the difference in MAE between two methods is statistically significant (S) or not significant (NS), according to the comparison criterion (Equation 7).

	RDKit-Direct	***RDKit-DFT***	***GOAT***	***openCOSMO-RS_c_p***
RDKit-Direct	—	S	S	S
RDKit-DFT	S	—	NS	NS
GOAT	S	NS	—	NS
openCOSMO-RS_c_p	S	NS	NS	—

Table S5: Pairwise statistical comparison of the MAEs reported in Figure 7, left, obtained for points whose molecules have < 5 rotatable bonds using the correlated composite uncertainty at the 95% confidence level. Each table entry indicates whether the difference in MAE between two methods is statistically significant (S) or not significant (NS), according to the comparison criterion (Equation 7).

	RDKit-Direct	***RDKit-DFT***	***GOAT***	***openCOSMO-RS_c_p***
RDKit-Direct	—	S	S	S
RDKit-DFT	S	—	S	S
GOAT	S	S	—	NS
openCOSMO-RS_c_p	S	S	NS	—

Table S6: Pairwise statistical comparison of the MAEs reported in Figure 7, right, obtained for points whose molecules have ≥ 5 rotatable bonds using the correlated composite uncertainty at the 95% confidence level. Each table entry indicates whether the difference in MAE between two methods is statistically significant (S) or not significant (NS), according to the comparison criterion (Equation 7).

	RDKit-Direct	***RDKit-DFT***	***GOAT***	***openCOSMO-RS_c_p***
RDKit-Direct	—	NS	NS	NS
RDKit-DFT	NS	—	NS	S
GOAT	NS	NS	—	NS
openCOSMO-RS_c_p	NS	S	NS	—

Table S7: Pairwise statistical comparison of the MAEs reported in Figure 8 with the full dataset ($N = 405$) using the correlated composite uncertainty at the 95% confidence level. Each table entry indicates whether the difference in MAE (solvation free energy) between two methods is statistically significant (S) or not significant (NS), according to the comparison criterion (Equation 7).

	RDKit-Direct	***RDKit-DFT***	***GOAT***	***openCOSMO-RS_c_p***
RDKit-Direct	—	S	S	S
RDKit-DFT	S	—	NS	S
GOAT	S	NS	—	S
openCOSMO-RS_c_p	S	S	S	—

Table S8: Pairwise statistical comparison of the RMSEs reported in Figure 8 with the full dataset ($N = 405$) using the correlated composite uncertainty at the 95% confidence level. Each table entry indicates whether the difference in RMSE (solvation free energy) between two methods is statistically significant (S) or not significant (NS), according to the comparison criterion (Equation 7).

	RDKit-Direct	***RDKit-DFT***	***GOAT***	***openCOSMO-RS_c_p***
RDKit-Direct	—	S	S	S
RDKit-DFT	S	—	S	NS
GOAT	S	S	—	NS
openCOSMO-RS_c_p	S	NS	NS	—

Boltzmann–Weighted Conformer Ensemble Calculations

For this exploratory study, the conformers were generated using the GOAT algorithm in a similar fashion as in the ***GOAT*** workflow, and subsequently relaxed at the DFT/BP86/def2-TZVP level in the gas phase. Due to computational limitations, the maximum number of conformers retained per compound was limited to 20, and this set was assumed to provide a representative sampling of the relevant conformational space accessible for each molecule. Then, for each conformer i and given solvent S, the free energy $G_i(S)$ was approximated as

$$G_i(S) = E_i(gas) + \Delta G_{i,solv}(S) \tag{1}$$

where $E_i(gas)$ is the gas-phase electronic energy and $\Delta G_{i,solv}(S)$ is the solvation free energy obtained from the openCOSMO-RS calculation. The solvent-specific Boltzmann weights for each conformer i were subsequently obtained at the target temperature T using

$$w_i(S) = \frac{e^{(-\Delta G_i(S)/RT)}}{\sum_{i=1}^{n} e^{(-\Delta G_j(S)/RT)}} \tag{2}$$

where $w_i(S)$ is the weight of conformer i in a specific solvent S, $\Delta G_i(S)$ is the relative energy of conformer i with respect to the lowest-energy conformer, R represents the ideal gas constant, and the summation $\sum_{i=1}^{n}$ includes the population n of retained conformers. This represents a practical estimate of solution-phase populations that does not account for explicit conformational entropy contributions beyond the computed free-energy terms.

For COSMO-RS solubility calculations, each conformer was treated as an independent species, yielding individual activity coefficients (γ_i) in the considered solvent. The calculated weights were thus used to compute solvent-dependent ensemble-averaged activity coefficients ($\gamma_{\text{ens}}(S)$) according to Equation 3[S2]

$$\ln \gamma_{\text{ens}}(S) = \sum_i w_i(S) \; \ln \gamma_i \tag{3}$$

where γ_i is the respective activity coefficient of conformer i in the considered solvent. The resulting ensemble activity coefficient ($\ln \gamma_{\text{ens}}(S)$) was subsequently employed in the solubility calculations, ensuring that conformational effects were accounted for and that the final solubility reflects the conformational ensemble rather than that of a single conformer.

References

(S1) Müller, S. Parameterization dataset openCOSMO-RS 24a. 2024; https://doi.org/10.5281/zenodo.13856201.

(S2) Klamt, A. *COSMO-RS: from quantum chemistry to fluid phase thermodynamics and drug design*; Elsevier, 2005.
